# Whole genome sequencing and phylogenetic analysis of strains of the agent of Lyme disease *Borrelia burgdorferi* from Canadian emergence zones

**DOI:** 10.1038/s41598-018-28908-7

**Published:** 2018-07-12

**Authors:** Shaun Tyler, Shari Tyson, Antonia Dibernardo, Michael Drebot, Edward J. Feil, Morag Graham, Natalie C. Knox, L. Robbin Lindsay, Gabriele Margos, Samir Mechai, Gary Van Domselaar, Harry A. Thorpe, Nick H. Ogden

**Affiliations:** 1 Genomics Core Facility, National Microbiology Laboratory, Public Health Agency of Canada, Canadian Science Centre for Human and Animal Health, 1015 Arlington St., Winnipeg, Manitoba Canada; 2 Zoonotic Diseases and Special Pathogens Division, National Microbiology Laboratory, Public Health Agency of Canada, Winnipeg, Manitoba Canada; 3 Department of Biology and Biochemistry, University of Bath, Claverton Down, Bath, United Kingdom; 4 Ludwig Maximilians Universität München, Department for Infectious Diseases and Zoonoses, Munich, Germany; 5 National Reference Centre for Borrelia, Oberschleissheim and Bavarian Health and Food Safety Authority, Oberschleissheim, Germany; 6 Public Health Risk Sciences Division, National Microbiology Laboratory, Public Health Agency of Canada, Saint-Hyacinthe, Québec, J2S 2M2 Canada

## Abstract

Lyme disease is emerging in southern Canada due to range expansion of the tick vector, followed by invasion of the agent of Lyme disease *Borrelia burgdorferi* sensu stricto. Strain diversity, as determined by Multi Locus Sequence Typing, occurs in this zone of emergence, and this may have its origins in adaptation to ecological niches, and have phenotypic consequences for pathogenicity and serological test performance. Sixty-four unique strains were cultured from ticks collected in southern Canada and the genomes sequenced using the Illumina MiSeq platform. A maximum likelihood phylogenetic tree of the chromosome revealed two large clades with multiple subclades. Consistent with previous studies on this species, the clades were not geographically defined, and some Canadian strains were highly divergent from previously sequenced US strains. There was evidence for recombination in the chromosome but this did not affect the phylogeny. Analysis of chromosomal genes indicated that these are under intense purifying selection. Phylogenies of the accessory genome and chromosome were congruent. Therefore strain differences identified in the phylogeny of chromosomal genes likely act as a proxy for genetic determinants of phenotypic differences amongst strains that are harboured in the accessory genome. Further studies on health implications of strain diversity are needed.

## Introduction

Lyme disease is a tick-borne zoonosis that in North America is caused by the bacterial pathogen *Borrelia* (or *Borreliella*) *burgdorferi* sensu stricto (henceforth shortened to *B. burgdorferi*). This bacterium is maintained in nature by wild animal reservoirs that live in woodlands^1^, and is emerging in eastern and central Canada due to northwards spread of the geographic range of the tick vector *Ixodes scapularis*^2^. The tick vector *Ixodes scapularis* and *B. burgdorferi* are species invading natural sylvatic communities in southern Canada, and the evolutionary history of *B. burgdorferi* associated with past expansions/invasions appears linked to phenotypes of public health importance^3^; both pathogenicity^4^ and detectability of infections by sero-diagnostic tests^5,6^ may vary with infecting strain. Whole genome sequencing (WGS) of bacterial pathogens has been successfully deployed in a public health context for high resolution genotyping, phylogeography, detecting emergent high-risk clones and source attribution^7,8^. The data can also be used to address fundamental evolutionary and ecological questions^9–12^. WGS of *B. burgdorferi* is set to also shed light on the public health risk in zones of emergence such as southern Canada by allowing us to identify sources and predict invasion patterns, and better understand the evolutionary ecology of *B. burgdorferi* and its associations with phenotype variations of ecological and public health significance^13^.

In recent years it has become recognised that *B. burgdorferi* is genetically diverse and a range of typing methods have been employed. Currently three main methods of typing are used because of their utility in identifying phenotypic traits such as pathogenicity and/or ecological, geographical and evolutionary patterns^14^. These are sequences of the 16S-23S *rrs-rrlA* intergenic spacer (IGS), *ospC* sequences, and multilocus sequence typing/multilocus sequence analysis (MLST/MLSA)^15^. IGS and *ospC* sequences are routinely clustered for strain typing using (i) Ribosomal Sequence Types (RSTs) or ribosomal spacer identification numbers (RSPs) for IGS^16,17^, and (ii) *ospC* major groups for *ospC*^18^. The diversity at *ospC* is believed to be maintained by balancing selection^18^ or niche specialisation^19^. Due to the focus on several housekeeping genes, phylogenetic trees constructed using the MLST sequences are likely to reflect the evolutionary history of *B. burgdorferi*, although there is considerable linkage disequilibrium in the *B. burgdorferi* genome and phylogenies constructed using IGS trees can show broadly similar patterns to those obtained by MLST trees. For *ospC*, however, the phylogenetic branching pattern was different from that of MLST genes suggesting differences in the evolutionary histories of these loci^15^.

MLST has provided data consistent with a south-to-north invasion of *B. burgdorferi* in Canada, in that many of genotypes detected in southern Canada are identical to those observed immediately to the south in northern USA^20^. However, in some parts of Canada (particularly southern Manitoba, north-west Ontario and the Maritime provinces) there are many diverse strains of *B. burgdorferi* in ticks and animal reservoir hosts, and some strains have to date only been found in Canada^20–22^. The possible ecological origins of this variation in strain diversity, and its consequences with respect to public health risk, specifically pathogenicity and serological test performance, are only just starting to be explored^3,23^.

Here we describe development and analysis of WGS data for 64 unique strains of *B. burgdorferi* collected in Canada in 2016. Phylogenetic analysis of strains from Canada, the US and Europe using WGS was compared against analyses obtained from sequences that are frequently currently used for strain typing and phylogenetic analysis of this species. Evidence for recombination, and its potential effect on strain phylogeny was explored. The strength and direction of selection across the chromosome was investigated to see if chromosomal genes could drive differences amongst strains in phenotypes of importance for public health (e.g., pathogenicity), that are reflected in the phylogeny. The sequence data we obtained will also provide a baseline resource for future studies of the evolutionary history of *B. burgdorferi*, and simultaneously allow exploration of what the observed diversity may mean for inter-strain phenotypic variations that are of public health significance.

## Results

### Samples collected and success of culture

**Figure 1:**
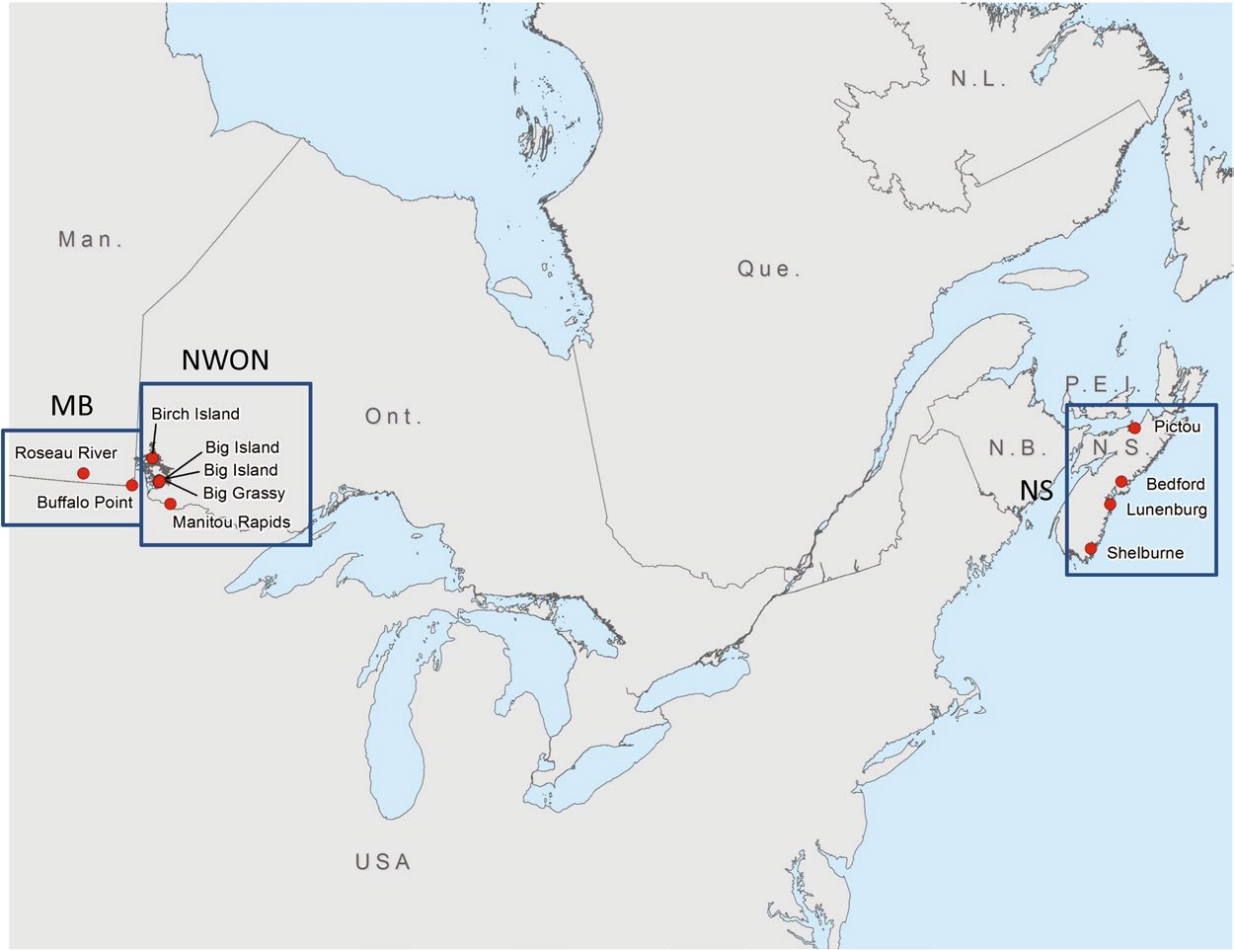
Location of sites from which host-seeking *I. scapularis* ticks were collected to obtain *B. burgdorferi* cultures. The circles indicate the groups of sample sites in southern Manitoba (MB), northwest Ontario (NWON), and Nova Scotia (NS). The Canadian provinces identified on the map are Man. = Manitoba, Ont. = Ontario, Que. = Quebec, N.L. = Newfoundland and Labrador, P.E.I = Prince Edward Island, N.B. = New Brunswick, N.S. = Nova Scotia. The map was created in ArcGIS Version 10.5 (ESRI, Redlands, CA: https://support.esri.com/en/products/desktop/arcgis-desktop/arcmap/10-5).

A total of 483 host-seeking blacklegged ticks were collected from 10 sites in Canada during October and November 2016 (Fig. 1), comprising two sites in Manitoba (Buffalo Point [n = 78] and Roseau River [70], MB), 4 sites in northwestern Ontario (Big Grassy [16], Big Island [11], Manitou Rapids [3], and Birch Island [12], ON), and 4 sites in Nova Scotia (in the counties of Bedford [60], Lunenburg [113], Pictou [34] and Shelburne [86], NS). From these samples, 64 unique single-strain cultures were obtained in the laboratory: 26 cultures from MB, 13 from ON, and 25 from NS (sample details are presented in Table S1). No cultures from solid media (the first step in the isolation procedure) contained more than one strain.

### Whole genomes sequenced

The chromosomes for all strains assembled as single contigs and were found to be collinear and between approximately 899 kb and 912 kb with a plasmid content ranging between 335 kb and 475 kb (Table S1). As previously reported^24^ the primary difference in chromosomal arrangement occurred at the 3′ (right) end. It is likely that the well documented repeat region in the *lmp1* gene on the chromosome^25^ contains inaccuracies. PCR performed using primers flanking this repeat region produced amplicons of variable size which were consistently larger than that predicted from the assemblies indicating that the repeat was likely collapsed during the assembly process. Unfortunately we were unable to resolve the exact number of repeats after conventional ABI sequencing. Estimates of PCR amplicon size ranged from approximately 950 bp–1600 bp corresponding to 4–8 copies. Because sequence data for all isolates assembled into a single, large, chromosomal contig; the remaining assembled contigs were considered to be plasmid content.

Using the paired end data, the strains were typed using the standard *Borrelia* MLST scheme. This revealed 27 different MLST Sequence Types (STs). There were 15 STs from MB, 7 STs from ON, 8 STs from NS. Of these, 7/20 (35%) STs from MB and NW ON are Canada-specific STs new to this study or found during recent studies^21,22^ (Table S1).

### Phylogenetic analysis

**Figure 2:**
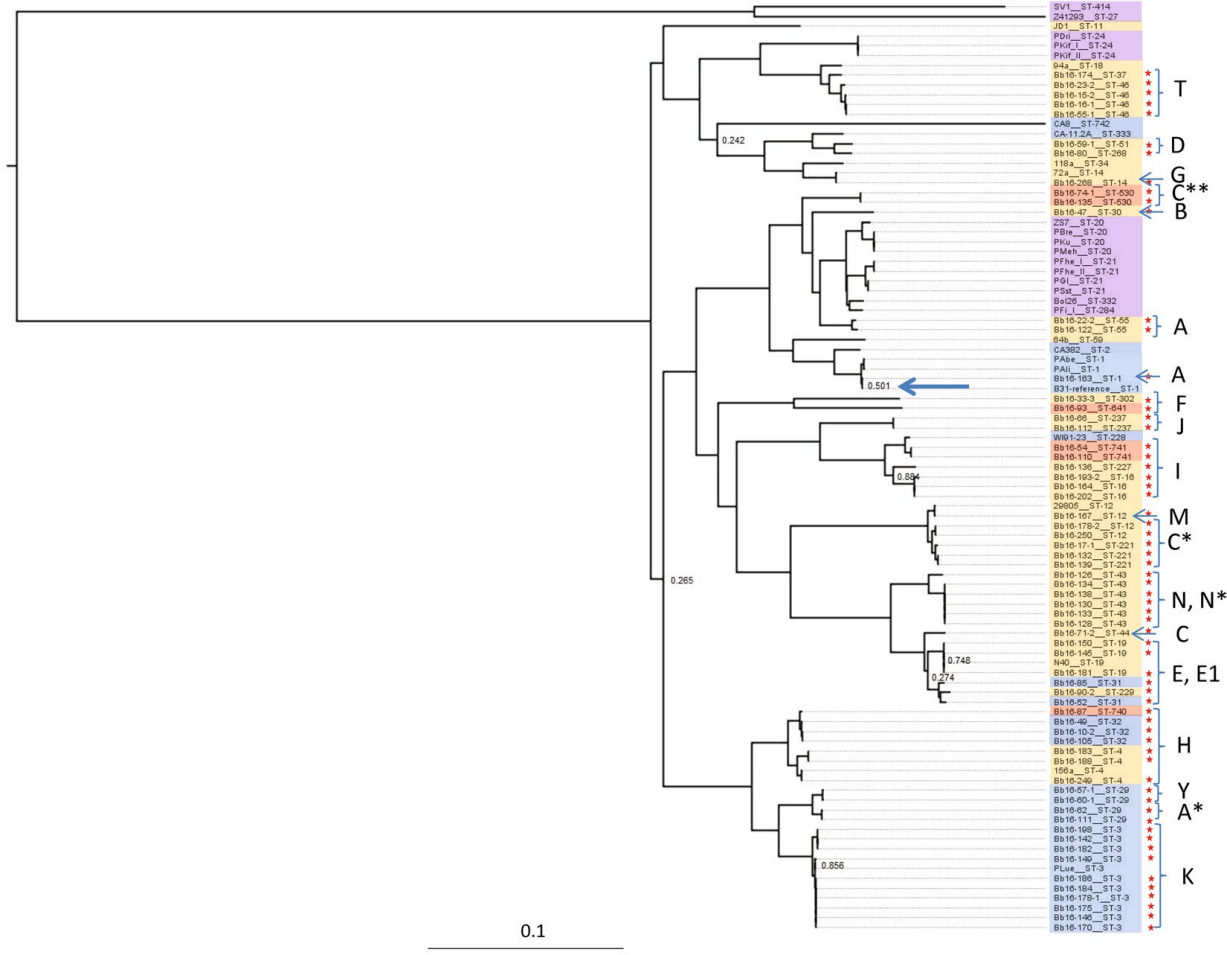
A midpoint rooted phylogenetic tree of the *B. burgdorferi* chromosome generated via the SNVPhyl pipeline. All sequences obtained in this study and currently available published full sequences from samples collected elsewhere in North America and Europe were used. Colour coding indicates the geographic occurrence of the MLST types of the sequences: blue = USA only; red = Canada only, yellow = USA and Canada; violet = Europe. Red stars to the right of the tree indicate sequences obtain from Canadian sites in this study. Letters to the right of the tree indicate the *ospC* major groups of the strains. Letters with asterisks indicate novel *ospC* major group sequences as described in the text. The position of the reference strain, B31 is shown by a blue arrow. Likelihood values for branches with a value less than 0.9 are shown.

The maximum likelihood phylogenetic tree (Fig. 2), constructed with the Canadian strains, and published sequences from Europe and the US (Table S2) comprises two well-supported major clades, within which are well-supported subclades. There is relatively high diversity of strains occurring in Canada, with sequences in almost all major and minor clades, as well as strains that cluster with the reference strain (B31). The clades and subclades in the phylogenetic tree are not defined geographically, with strains from Canada and the US in most. Two clades of European strains nested within clades containing strains from North America. Some Canada-specific strains are very similar to other known strains but some are quite divergent (Fig. 2). A similar phylogenetic tree was obtained using some of the sequences from Walter *et al*.^26^, although some of the new sequences from this study did cluster with the divergent Canadian strains (Fig. S1).

Strains of the same MLST ST always clustered together in the whole chromosome tree (Fig. S2) and the tree constructed only using the MLST sequences formed the same major clades as the whole chromosome tree with some minor differences (Fig. S3). Analysis of outer surface protein C (*ospC*) sequences revealed that the Canadian strains collectively carried 14 previously described *ospC* major groups (A, B, C, D, E, E1, F, G, H, I, J, K, M, N). In addition, 11 strains carried *ospC* sequences that had similarities to existing major group sequences that were too low (<90%) to be classified as belonging to an existing *ospC* major group. These sequences have been temporarily named according to the closest major group as A*, C*, C** and N* (Figs 2 and S4). The strains carried IGS sequences determined as belonging to 12 IGS genotypes: 1A, 2A, 2D, 4, 4A, 5, 6A, 6B, 7A, 7B, 8C and 9 (Fig. S5). The clade structures in the trees of the *ospC* and IGS sequences had a greater number of differences than the MLST tree when compared to the whole chromosome tree (Figs S4 and S5).

### Evidence of strong purifying selection on the chromosomal genes

**Figure 3:**
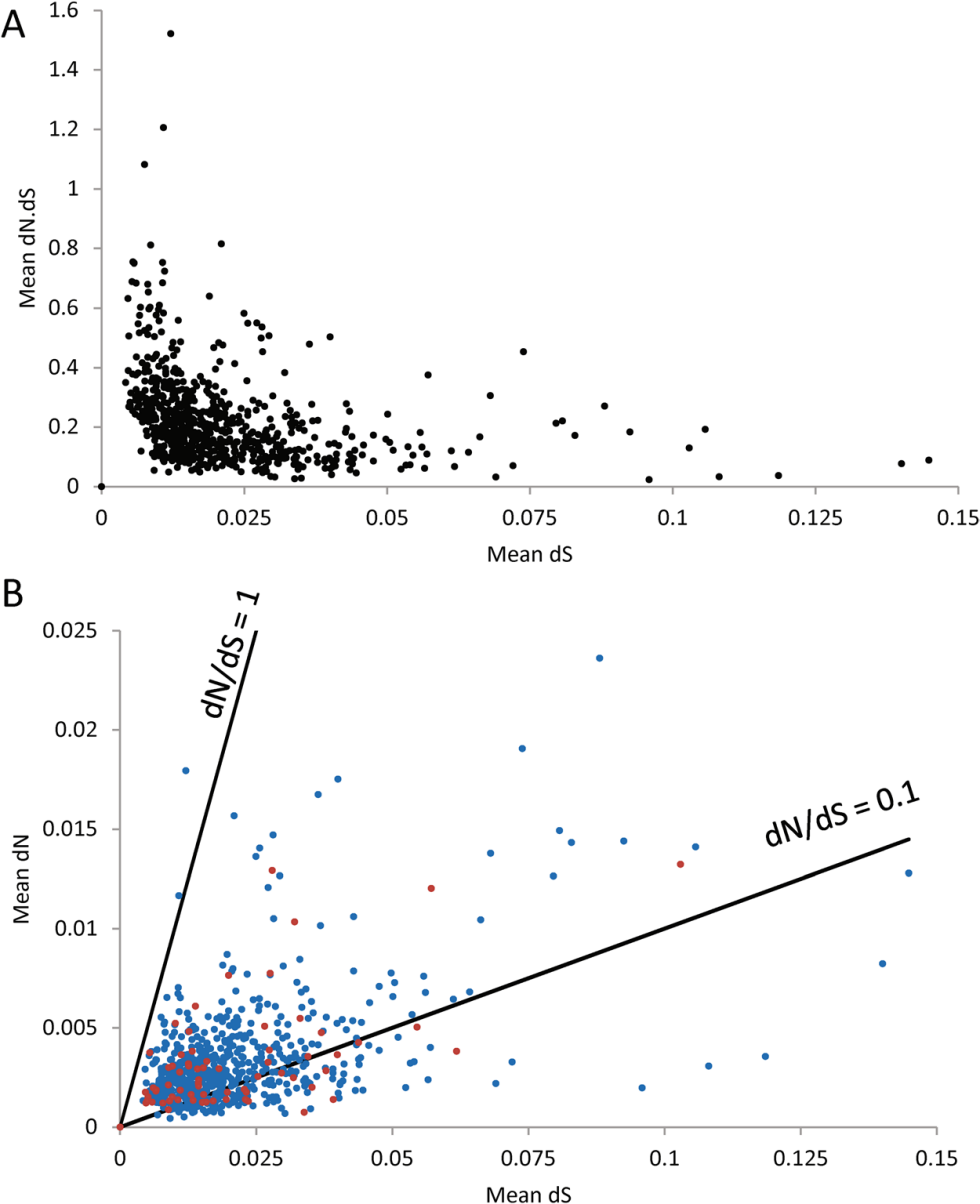
Output of assessment of strength and direction of selection on chromosomal genes. (**A**) A plot of pairwise dN/dS versus divergence time (dS) over all chromosomal genes. (**B**) A plot of dN versus dS for all chromosomal genes. Those genes encoding surface exposed proteins, or other genes which are putatively immunogenic (see Table S2) are shown in blue, all other genes are shown in red. The lines represent two interpretations of the neutral expectation (dN/dS = 1 and 0.1). The single gene with dN/dS > 1 is rpsH which encodes a ribosomal protein hence would be expected to be under strong stabilising selection.

In order to examine the strength and direction of selection on the chromosomal genes we calculated dN and dS for all genes in turn using the method of Nei & Gojobori^27^. Non-syonymous changes are on average more likely to be deleterious than synonymous changes, hence the dN/dS ratio provides evidence as to the strength of purifying selection (where dN/dS < 1) or positive selection (dN/dS > 1). We first examined the trajectory of dN/dS over divergence time (dS). Purifying selection operating over time will lower dN/dS at a rate determined by the selection co-efficients of the non-syonymous changes (*s*) and the effective population size (*N*_*e*_)^28^. Figure 3a shows pairwise dN/dS against dS over all chromosomal genes. Although there is weak evidence of a slight increase in dN/dS over very short divergence times (low dS), in general dN/dS is very low (~0.1) regardless of dS. This points to very strong purifying (stabilising) selection acting on the chromosomal genes as a whole.

We then considered whether we could detect any single genes for which there was evidence of positive rather than purifying selection by plotting, for each gene, dN/dS (Fig. 3b), and comparing these ratios with the neutral expectation (dN/dS = 1). We separately considered all those genes listed in Table S3 which are surface exposed or else putatively immunogenic. These genes might be expected to be under positive selection, in which case we might expect dN/dS > 1. Figure 3b reveals that for all genes, including those encoding surface exposed proteins (shown in blue) fall well below the neutral expectation (dN/dS = 1), which is consistent with strong purifying selection across the chromosome. The only possible exception is the *rpsH* gene (locus tag BB0493), which encodes the 30S ribosomal protein S8, which has an overall dN/dS > 1. As there is no obvious reason why this gene should be subject to positive selection, this analysis again points to very strong stabilising selection acting on the chromosomal genes. To support this further, we note that there are only 11 nonsense mutations in the data, out of a total of 13,507 SNPs (synonymous = 8463, non-synonymous = 4193, intergenic = 903). Thus only 0.081% of the total SNPs potentially resulted in a loss of gene function, which is approximately half the proportion we observed in comparable datasets for *S. aureus* (0.19%) and *E. coli* (0.19%). This suggests that a high proportion of the genes retained on the chromosome are essential, which again is consistent with strong purifying selection. It has been suggested that, for within-species inter-strain comparisons, a threshold dN/dS value of 0.1 rather than 1 may be more appropriate for identifying genes under positive selection^29^. There were 574 genes with dN/dS values above this threshold, but the proportion of genes likely encoding surface-exposed proteins above the threshold (53/74, 71.6%) was not significantly different from that for genes that likely encode non-surface-exposed proteins (521/674, 77.3%, χ^2^ = 1.2, degrees of freedom = 1, *P* > 0.1).

### Evidence for recombination in the sequences

**Figure 4:**
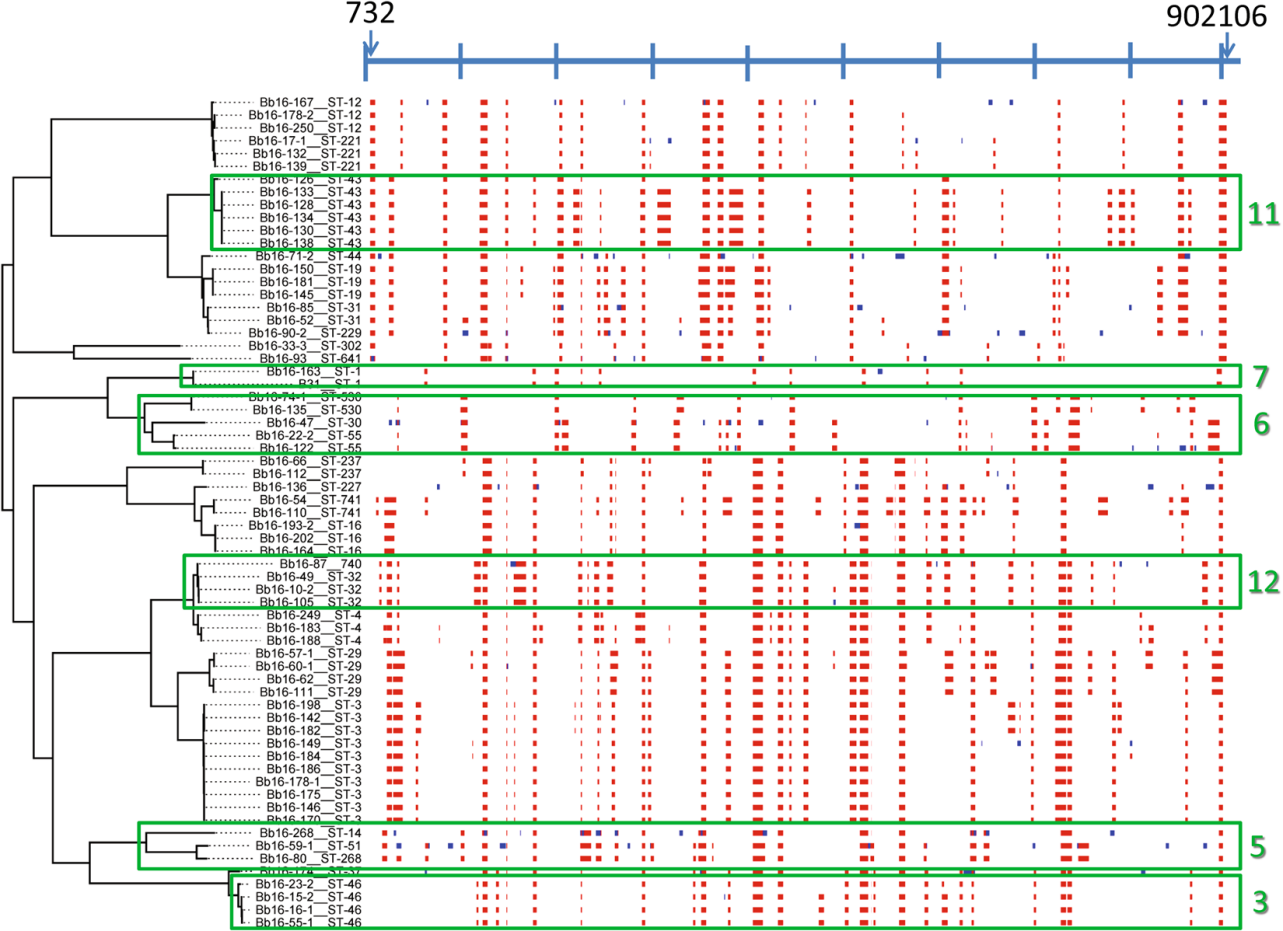
Output from Gubbins analysis showing a map of possible regions of recombination. The scale bar at the top of the map shows the length of the chromosome (with tick marks every 100 Kb) and arrows indicate the first and last position where recombination events were detected. The analysis included all sequences obtained in this study. Red regions are those that are common to multiple strains, the blue regions are those found only in one strain, and white areas indicate where recombination was not detected. Green boxes illustrate some of the clades that had patterns of recombination loci that were similar within-clade but different to adjacent clades. The numbers refer to the numbering of clades in the phylogenetic tree in Fig. 6.

**Figure 5:**
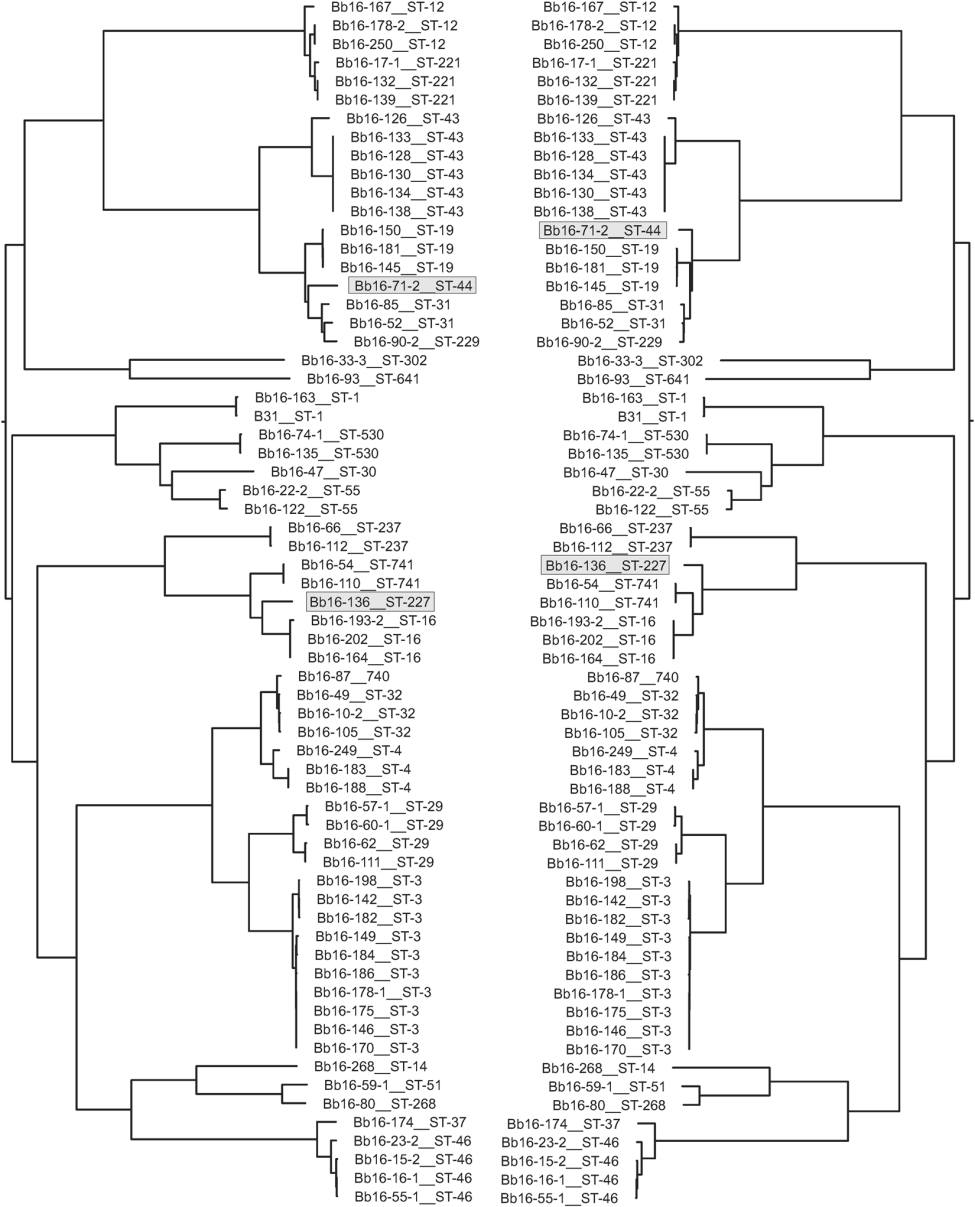
A comparison of phylogenies with and without potential recombinant regions. Phylogenetic tree of the *B. burgdorferi* ss chromosome generated via the SNVPhyl pipeline is shown on the left and a tree constructed using Gubbins after exclusion of potential recombination regions is shown for comparison on the right.

**Figure 6:**
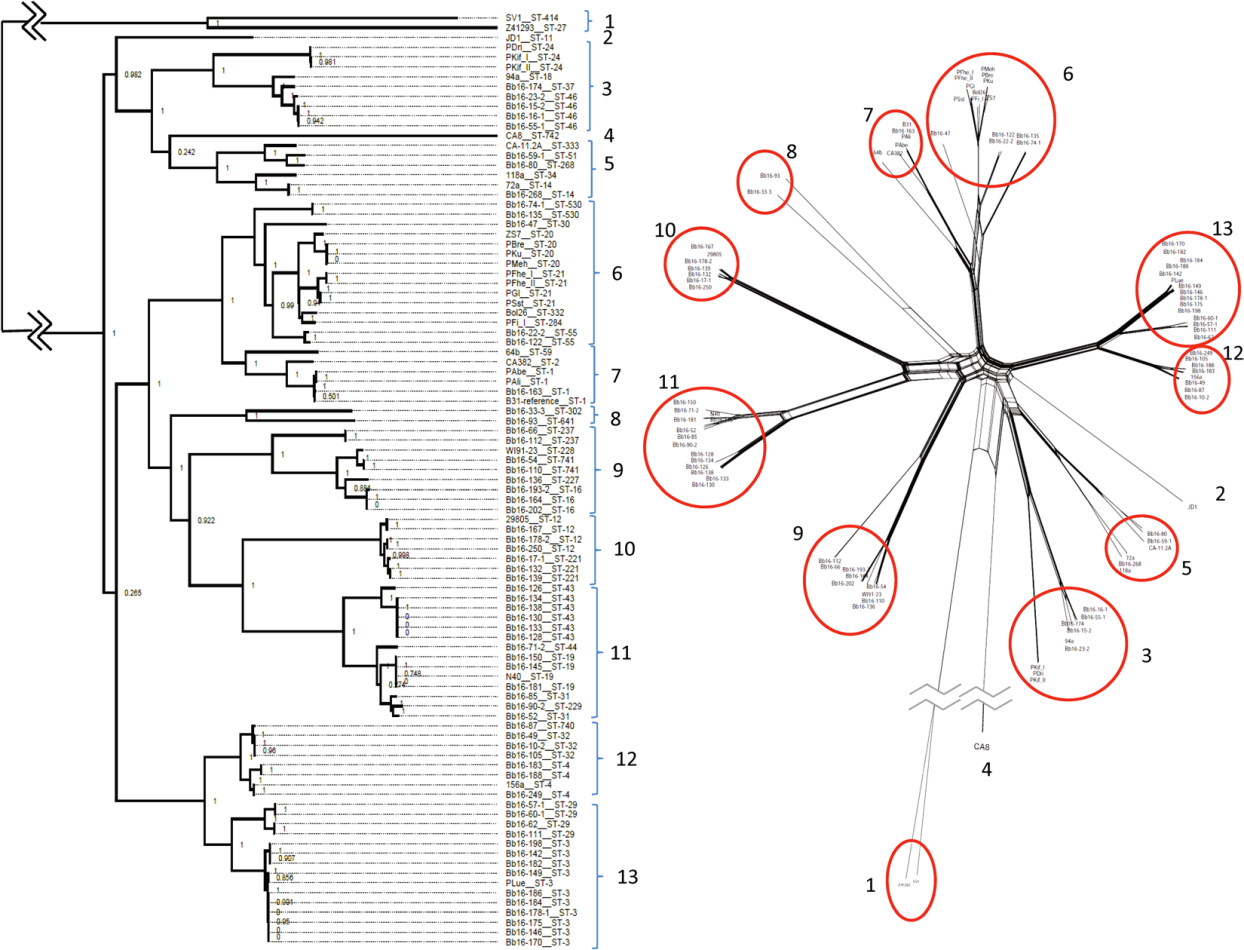
A comparison of clades identified by maximum likelihood via the SNVPhyl pipeline and by network analysis. The orientation of clades and divergent single strains are identified by brackets in the maximum likelihood phylogenetic tree (left) and clusters are identified by red circles in the network analysis (right). The numbers identify the clades and corresponding clusters.

Recombination analysis using Gubbins^30^ identified potential regions of recombination occurring throughout chromosome, with 61% of chromosomal genes being implicated in recombination in at least one strain (Fig. 4). The PhiTest implemented in SplitsTree4 (version4.1.3.1)^31^ detected statistically significant evidence for recombination for the chromosome, whether or not *B. bissettiae* strain DN127 was used as outgroup (Table 1). There was a mean 3.58 recombination events per branch and the mean ratio of SNPs introduced by recombination to those introduced by point mutations was 1.12. The regions of recombination along the chromosome are similar amongst strain of the same clade but more different in strains in different clades (Figs 4 and S6). Excluding regions of recombination identified by Gubbins from the phylogenies did not significantly alter the phylogenetic relationship among the strains as determined by the core genome SNP analysis (Fig. 5). Similarly, strains that clustered in the phylogenetic tree also clustered in the NeighbourNet network analysis (Fig. 6).

**Table 1:** Results of the PhiTest for evidence for recombination for the chromosome using SplitsTree.

	With *B. bissettiae* as outgroup	Without outgroup
Informative sites	23953	22542
Window	100 with k as 38	100 with k as 69
Mean	0.243	0.170
Variance	1.338 × 10^−7^	5.741 × 10^−8^
Observed	0.203	0.144
*P*	<0.001	<0.001

### A highly variable accessory genome

**Figure 7:**
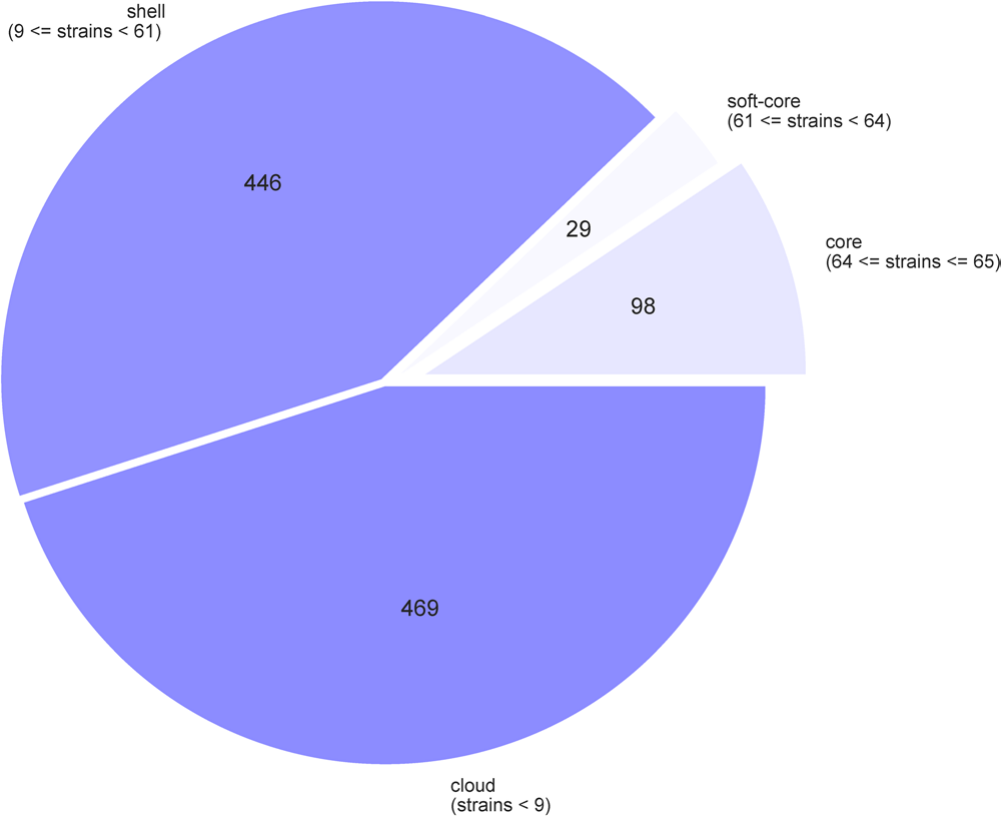
Pie chart of the breakdown of plasmid genes and the number of isolates in which they are present.

**Figure 8:**
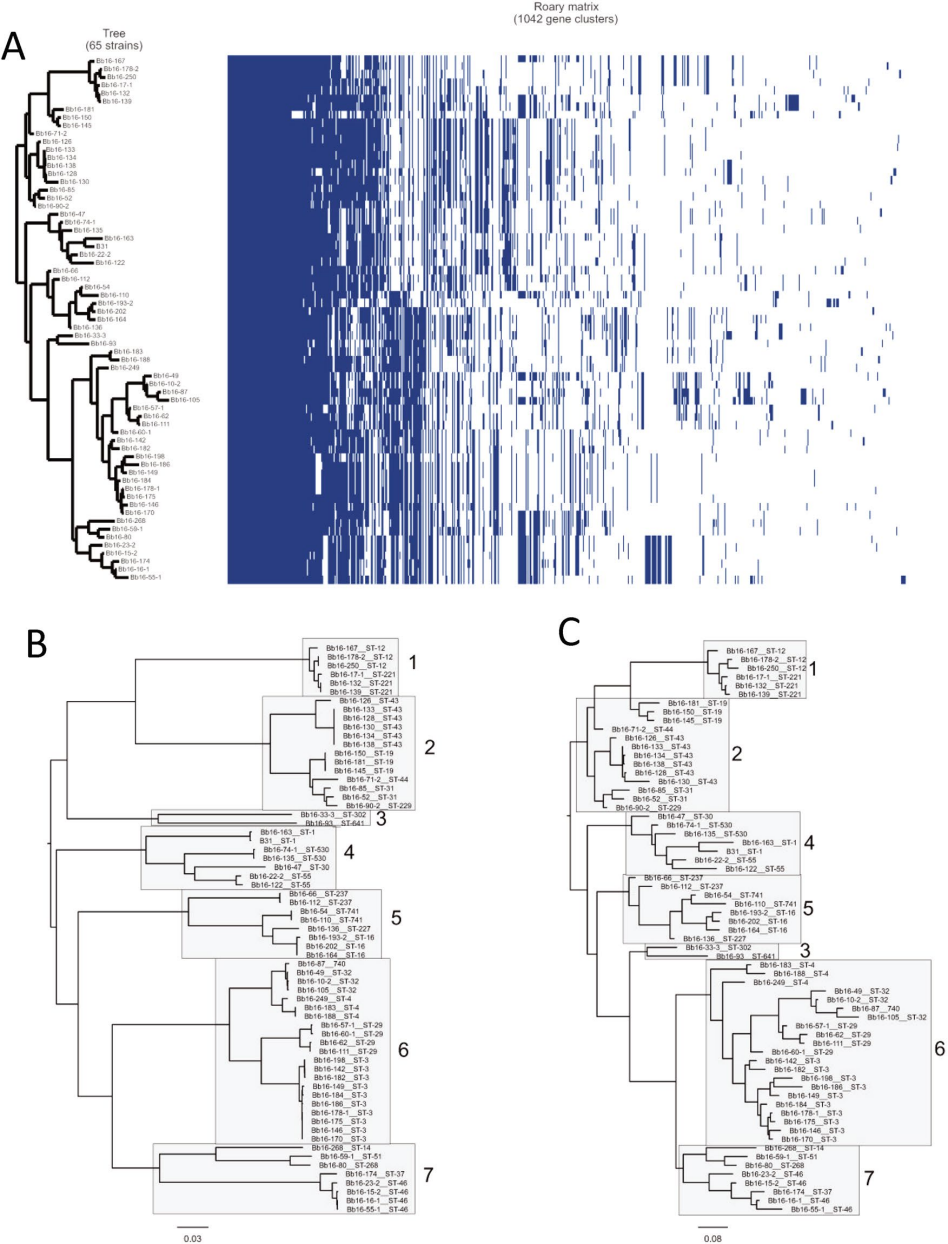
Similarity in phylogeny generated from the chromosome and the accessory genome. (**A**) Phylogenetic relationship of plasmid genome as determined by Roary compared to a matrix with the presence (blue blocks) and absence (grey areas) of core and accessory genes found in the pangenome. Correlation between clades as determined by (**B**) SNP analysis of the chromosome and (**C**) pangenome analysis of the plasmid genome is illustrated. The relationship among the strains is consistent between the two methods although the absolute placement of the clade denoted as #3 differs.

The accessory genome was found to be highly variable among the isolates. Of the 1042 gene clusters identified by Roary^32^ only 127 were found to be present in greater than 95% of the isolates, 446 of the clusters fell between 15% and 95% of the isolates and 469 were present in 15% or less (Fig. 7). However, the clustering of the strains based on the presence or absence of these genes was consistent with that found via SNP analysis of the chromosome, with the exception of a difference in the position in the tree of one clade (Fig. 8).

## Discussion

At the time of writing, in this study we have more than doubled the currently available high quality whole genome sequences of *B. burgdorferi*. The data provided by these sequences will provide a resource for study of phylogenetics and functional genomics of this bacterium (e.g. mechanisms of host associations, strain-specific pathogenicity, strain-specific detectability by diagnostic tests) in the future. The high yield of single-strain cultures using the semi-solid phase plating technique demonstrated its utility for obtaining cultured strains from field-collected samples for WGS.

The sequences obtained were concordant in structure to those previously sequenced (Table S2) with sequences being assembled into a single chromosomal contig, a large number of sequences forming the accessory genome, greater inter-strain variation at the 3′ end of the chromosome^33^ and variation in length associated with a variable-length repeat region^25^.

The phylogenetic tree obtained from the whole chromosome is consistent with previous trees made using MLST^15,22^ in that there is a strong clade structure but this is not based on the geographic origin of strains (i.e. isolation by distance). However, the WGS provided trees of finer precision in identifying strain differences, by having better support of internal branches, than any method used to date. It should be noted that one clade of strains (comprising strains PAli, PAbe und PLue) that was found in European patients resemble North American strains. These strains have never been found in ticks in Europe and it has been suggested that the affected people acquired their infection in North America^34^. There was evidence for recombination in the chromosome (which occurred at rates similar to those in other studies)^26^ but this had only very slight impact on the structure of the phylogenetic tree, which may be due to recombination events occurring in similar regions within-clades, but in more different regions between clades. This suggests that both point mutations and recombination events have occurred contemporaneously in broad terms during the population processes that drove development of the clades.

Some of these Canada-specific strains are very similar to strains occurring in the US. However some Canada-specific strains (e.g. 96, 74-1 and 135: Fig. 2) are quite divergent from the strains occurring in the US for which full sequences are available. Strains 74-1 and 135 also carried a novel *ospC* allele (Fig. 2). We have in the past suggested that strains novel to Canada are not from refuges in Canada, but represent strains endemic to the northern US that have yet to be discovered there^3^. Consistent with this, some strains from ticks in the northern US identified by Walter *et al*.^26^ clustered with the divergent Canadian strains (Fig. S1). The WGS supports previous studies using MLST in terms of (i) the topology of the phylogenetic tree, which was similar to the tree generated using MLST sequences alone, and in which strains of the same MLST type always clustered together, and (ii) in the identification of strains (and MLST types) that are (to date) unique to Canada. The study also found some of the Canada-specific MLST STs that we identified in recent studies^20–22^ confirming the reproducibility of this work. The ecological and evolutionary origins of the tree structure continue to be intriguing and will continue to be a focus for research.

Of the 64 unique strains, 22 carried *ospC* alleles of major groups considered as being particularly associated with disseminated Lyme disease (A, B, I, K and N: reviewed in^35^). To what extent the other *ospC* sequences, and the new *ospC* sequences are associated or not with disseminated disease, and where they occur, remains to be studied in Canada.

There was evidence for very strong stabilising selection acting on chromosomal genes, which suggests that the vast majority of adaptive changes occur within the much more dynamic accessory genome carried on the plasmids. The distinction between the core and accessory genomes in *B. burgdorferi* is therefore particularly striking as these two components are physically unlinked. In this way the bacteria can maintain essential genes on the chromosome, which are subject to strong purifying selection and only low rates of recombination, whilst ecological adaptations are made possible largely through the much more “sexual” accessory genome borne on the plasmids. This suggests that associations between strain type and phenotypic characteristics (interactions with different host species^22^, pathogenicity^4^, and possibly the detectability of antibody responses to some surface proteins by serological tests)^6^ are mostly driven by genetic variations in the accessory genome rather than by those in the chromosome. The phylogenies of the accessory/plasmid content and the chromosome were consistent (as in other recent studies)^36^ so strain differences identified in the phylogeny of chromosomal genes likely act as a proxy for the genetic determinants of phenotypic differences that are mostly in the accessory genome. This is consistent with our previous conclusions regarding associations of MLST types with phenotypic differences^4,22^. However, the plastic nature of plasmid content amongst the strains means that even strains that are phylogenetically closely related could harbor differences in gene content that have important impacts on the phenotypes.

The analysis of the accessory genome is consistent with previous studies showing that plasmids within the genospecies *B. burgdorferi* show a high degree of diversity^36,37^. Although some linear plasmids can be assembled from short reads only, long-read methods such as Pacific Bioscience SMRT technology or Nanopore Sequencing technology are likely to be required to explore the plasmid content of *Borrelia* strains in detail^38^.

In this study we have sequenced the genome of 64 strains of *B. burgdorferi* s.s. adding considerably to our data on, and knowledge of, this bacterium. These data will help us explore questions regarding pathogenicity, ecology and epidemiology that are relevant for public health responses to the increasing threat from Lyme disease.

## Materials and Methods

### Collection and selection of samples for culture

Samples for the study comprised host-seeking ticks collected by drag sampling^39^ from areas of *I. scapularis* and *B. burgdorferi* invasion in south central Canada (southeastern Manitoba [MB] and northwestern Ontario [ON]) and the Maritimes (Nova Scotia [NS]) (Fig. 1). Ticks collected from the environment were kept alive at room temperature by placing 15 ml vials containing ticks in plastic storage bags provisioned with moistened paper towel to maintain humidity over 90%. Ticks were transported by surface transport (from Manitoba and northwestern Ontario) or courier (from Nova Scotia) to the National Microbiology Laboratory in Winnipeg. At the laboratory, ticks were surface decontaminated by immersion in the following sequence of solutions prepared with sterile nuclease-free water; 0.5% bleach containing one drop of Tween 80 per 10 ml, 0.5% benzalkonium chloride, 3% hydrogen peroxide, and 70% ethanol, and then rinsed in nuclease free water. Ticks were disinfected individually or in pools of up to ten specimens if collected from the same site. Individual ticks were transferred to sterile 1.5 ml tubes containing Barbour-Stoenner-Kelly (BSK) medium prepared in-house and supplemented with rifampicin, kanamycin, phosphomycin, amphotericin B, and heat-inactivated rabbit serum (Millipore Sigma, Oakville, Canada). Ticks were then cut into small pieces using a sterile scalpel and a 100 µl aliquot of the tick macerate was removed for DNA extraction using a Qiagen DNeasy 96 kit. Extracted DNA was tested by real-time PCR targeting the 23S rRNA gene of *B.burgdorferi* s.l.^40^. The remaining material was held at 4 °C until PCR was complete and samples that were positive for *B. burgdorferi* by PCR were cultured in semi-solid agar.

### Semi-solid phase plating for recovery of single-strain colonies

In our experience, approximately 12% of ticks from collection sites in Canada show evidence of mixed-strain infections^22^. In order to obtain clonal populations of single strains of *B. burgdorferi*, tick homogenates were cultured in semi-solid agar. Undiluted, 10^−1^ and 10^−2^ dilutions of tick homogenates were added to a solution of BSK and 0.7% agarose held at 42 °C. The mixtures were then poured into plastic petri plates and allowed to solidify. Plates were incubated at 37 °C with 5% CO_2_ for up to 28 days. Two detectable colonies per tick were excised from the agar and seeded into separate vials containing liquid BSK-H (Millipore Sigma, Oakville, Canada) for expansion of the culture at 35 °C for 7 day*s*. Once cultures were in exponential phase of growth, an aliquot was removed for DNA extraction for genome sequencing. The remaining sample was centrifuged and resuspended in cBSK supplemented with 10% glycerol for cryopreservation in liquid nitrogen.

### Genome sequencing and analysis

Multiplexed libraries were created with TruSeq sample preparation kits (Illumina, San Diego, CA) and paired-end, 300 bp, indexed reads were generated on the Illumina MiSeq platform (Illumina, San Diego, CA). Genome assembly was performed using SPAdes v3.9^40,41^ with a minimum contig size cut off of 1000 bp.

For MLST determination, paired end reads for each isolate were directly compared to the eight housekeeping loci (*clpA*, *clpX*, *nifS*, *pepX*, *pyrG*, *recG*, *rplB*, and *uvrA*) for other *B. burgdorferi* sensu stricto isolates in the MLST database at the University of Oxford (http://www.pubmlst.org) using SRST2 v.0.3.6. Novel MLST types found in this study were submitted to the database curators for verification, classification, and inclusion in the database.

### Phylogenetic analyses

A maximum likelihood phylogenetic tree of the bacterial chromosome was created with SNVPhyl, a whole genome SNV phylogenomics pipeline^42^ using B31 (Table S2) as the reference sequence, which was originally isolated from a tick in northeastern US^43,44^. The analysis was performed with all strains previously described^33^ from Europe and the USA excluding two European strains (PHas and PMi), which were omitted due to the low coverage present in the SRA data. In cases where the raw reads were unavailable these were simulated based on the WGS scaffolds using ART^45^.

Recently a large number of sequences of the *B. burgdorferi* genome obtained from ticks collected in North America have been published^26^. However these sequences were obtained by a hybrid capture method that has some limitations in terms of sequence quality that make these sequences incompatible for analysis with those generated here. These include low coverage in approximately 10% of strains, absence of sequences that are not present in the reference strain B31 (which is used to assemble the reads), an unknown number of strains that have chimeric sequences that are obtained because the method obtains reads from all strains in mixed infections (which were found in about 50% of the ticks), as well as the possibility of chimeras that include sequences of genes of other bacteria species present in the ticks, where these are conserved across bacterial species^26,46^. Nevertheless, we selected sequences from this study that we consider the least ambiguous (as described in supplementary information), to include in a maximum likelihood phylogenetic tree (also created with SNVPhyl) with our own sequences and previously published sequences to see if their inclusion markedly alters the phylogeny we obtained without them.

A Bayesian phylogenetic tree using the MLST sequences^15^ of the strains was constructed using MrBayes v3.2.1^47^ to compare the tree topology obtained against the tree generated from the whole chromosome. Markov chain Monte Carlo (MCMC) samplings were run for 500,000 generations with trees being sampled every 1,000th generation, and convergence diagnostics conducted as previously described)^47–49^.

Because *ospC* – coding sequences (which occur on the circular cp26 plasmid), and the chromosomal 16S–23S *rrs-rrlA* IGS sequences are frequently used in strain typing of *B. burgdorferi*, these sequences were obtained and analysed as follows. To obtain the *ospC* sequences for each strain, paired end reads were mapped to the B31 reference strain including plasmid sequences. The consensus for the *ospC* gene from plasmid cp26 (nucleotides 16903 through 17535 as per AE000792.1 for a total of 632 bp), obtained as described below, was pulled out for each isolate. The sequences were aligned using ClustalW2 (v2.1, EMBL-EBI, Hinxton, Cambridgeshire, UK) and typed to *ospC* major groups using the similarity criteria of Qiu *et al*.^18^. The IGS region for each isolate was extracted in an identical manner as that for *ospC* based on annotation from the B31 reference chromosomal sequence. Sequences were aligned, trimmed, and genotyped according to the criteria of Bunikis *et al*.^50^. Bayesian phylogenetic trees of the *ospC* and IGS sequences were also constructed using MrBayes v3.2.1 for comparison against the tree generated from the whole chromosome of the strains.

### The strength and direction of selection on the chromosomal genes

We examined the degree to which the purifying and positive selection is operating on chromosomal encoded genes by plotting dN (the number of non-syonymous SNPS per non-syonymous site) against dS (the number of synonymous SNPs per synonymous site). dN and dS were calculated using the method of Nei and Gojobori^27^. In order to detect whether potentially immunogenic genes were under unusually strong positive selection, we separately considered surface expressed proteins, and/or proteins included in the two-tier serological test for Lyme disease^51^, and compared these against all other chromosomal genes. The proteins are listed in supplementary information Table S3.

As dN/dS has also been shown to decrease with increasing divergence (dS) in bacteria, owing to ongoing purifying selection of slightly deleterious non-synonymous changes^28^, we also plotted pairwise dN/dS against dS using averages of all chromosomal genes.

### Assessment of the degree of recombination in the sequences

An alignment was generated by incorporating the variable sites identified in each strain via the SNVPhyl analysis using the B31 sequence as the reference sequence using in-house scripts. This was then used to assess the effect of recombination on the phylogenies using Gubbins. The PhiTest implemented in Split Tree 4 software was also used to evaluate phylogenetic networks based on the alignment. Multiple alignments of strains (from this and previous studies, Table S1) in Phylip were uploaded and a network analysis (NeighborNet) was conducted in Splitstree4^31^ using standard settings. The Phi Test was conducted using *B. bissettiae* strain DN127 as outgroup or with only *B. burgdorferi* strains.

### Analysis of the accessory genome

The plasmid content among the strains was compared by first assembling the genomes using SPAdes v3.9^41^ with a minimum contig size cut off of 1000 bp. The chromosomal contigs were removed from the collection for each strain and those remaining were assessed via a pangenome approach using Roary^32^ allowing for the collapse of paralogs. All 21 complete plasmid sequences associated with the B31 reference sequence were included (Table S1). Phylo.io^52^ was used to compare the resulting phylogenetic tree, based on gene possession in the accessory genome, to the one based on the chromosome generated using SNVPhyl.

### Data availability

All data have been submitted to Genbank under BioProject PRJNA416494, while the MLST and isolate data are available at the MLST website http://pubmlst.org/borrelia.

## Electronic supplementary material

Supplementary information: Shaun Tyler, Shari Tyson, Antonia Dibernardo, Michael Drebot, Edward J Feil, Morag Graham, Natalie C. Knox, L Robbin Lindsay, Gabriele Margos, Samir Mechai, Gary Van Domsela
