# Supplementary material for: Whole genome sequencing and phylogenetic analysis of strains of the agent of Lyme disease Borrelia burgdorferi from Canadian emergence zones
Source: Sci Rep. 2018 Jul 12;8:10552. doi: 10.1038/s41598-018-28908-7 (PMC6043495; doi:10.1038/s41598-018-28908-7)
Supplement: Supplementary file 1 — Supplementary information: Shaun Tyler, Shari Tyson, Antonia Dibernardo, Michael Drebot, Edward J Feil, Morag Graham, Natalie C. Knox, L Robbin Lindsay, Gabriele Margos, Samir Mechai, Gary Van Domsela [file 41598_2018_28908_MOESM1_ESM.pdf]

Supplementary information: Shaun Tyler, Shari Tyson, Antonia Dibernardo, Michael Drebot, Edward J Feil, Morag Graham, Natalie C. Knox, L Robbin Lindsay, Gabriele Margos, Samir Mechai, Gary Van Domselaar, Harry A Thorpe, Nick H Ogden. Whole genome sequencing and phylogenetic analysis of strains of the agent of Lyme disease *Borrelia burgdorferi* from Canadian emergence zones

Table S1. The samples from which single-strain cultures and whole genome sequences were obtained. The deduced MLST sequence types (ST) is shown. Double asterisks indicate STs new to this study and single asterisks indicate STs found only in Canada to date that have been found in previous studies. MB = Manitoba, NS = Nova Scotia, NWON = northwest Ontario, CON = central Ontario; F = female and M = male adult *Ixodes scapularis* ticks. Chr bp = number of base pairs in the chromosome, Plas bp = the number of base pairs in the plasmids.

Sample ID	Tick	Province	Sampling site	Site latitude	Site longitude	Chr bp	Plas bp	ST
Bb16-105	F	MB	Buffalo Point	49.0127	-95.2396	909754	384626	32
Bb16-110	F	MB	Buffalo Point	49.0127	-95.2396	902945	377837	740**
Bb16-111	F	MB	Buffalo Point	49.0127	-95.2396	903043	397199	29
Bb16-112	F	MB	Buffalo Point	49.0127	-95.2396	902747	383914	237
Bb16-17.1	F	MB	Buffalo Point	49.0127	-95.2396	902885	391924	221
Bb16-22.2	M	MB	Buffalo Point	49.0127	-95.2396	909902	420793	55
Bb16-23.2	M	MB	Buffalo Point	49.0127	-95.2396	910005	390019	46
Bb16-49	F	MB	Buffalo Point	49.0127	-95.2396	909772	398657	32
Bb16-52	F	MB	Buffalo Point	49.0127	-95.2396	902473	396242	31
Bb16-54	M	MB	Buffalo Point	49.0127	-95.2396	903176	407735	741**
Bb16-55.1	M	MB	Buffalo Point	49.0127	-95.2396	910063	381025	46
Bb16-57	M	MB	Buffalo Point	49.0127	-95.2396	912601	383209	29
Bb16-59	M	MB	Buffalo Point	49.0127	-95.2396	909679	450144	51
Bb16-60	M	MB	Buffalo Point	49.0127	-95.2396	912332	394463	29
Bb16-62	M	MB	Buffalo Point	49.0127	-95.2396	900013	393463	29
Bb16-66	M	MB	Buffalo Point	49.0127	-95.2396	902716	371215	237
Bb16-71.2	M	MB	Buffalo Point	49.0127	-95.2396	899098	410128	44
Bb16-74.1	M	MB	Buffalo Point	49.0127	-95.2396	910366	413495	530*
Bb16-80	M	MB	Buffalo Point	49.0127	-95.2396	909757	426078	268
Bb16-85	M	MB	Buffalo Point	49.0127	-95.2396	902830	405435	31
Bb16-87	M	MB	Buffalo Point	49.0127	-95.2396	905868	353188	740**
Bb16-90	M	MB	Buffalo Point	49.0127	-95.2396	899030	395326	229
Bb16-93	M	MB	Buffalo Point	49.0127	-95.2396	902774	423999	641*
Bb16-10.2	F	MB	Roseau River	49.2013	-96.9102	909609	384544	32
Bb16-15.2	F	MB	Roseau River	49.2013	-96.9102	909692	378059	46
Bb16-16.1	F	MB	Roseau River	49.2013	-96.9102	910101	362448	46
Bb16-122	M	NWON	Big Grassy	49.1002	-94.3276	909992	425481	55
Bb16-126	F	NWON	Big Grassy	49.1002	-94.3276	902186	384995	43
Bb16-128	F	NWON	Big Island	49.1455	-94.3146	902646	375753	43
Bb16-130	F	NWON	Big Island	49.1215	-94.3146	902435	343897	43
Bb16-133	M	NWON	Big Island	49.1215	-94.3146	902882	371382	43
Bb16-134	M	NWON	Big Island	49.1215	-94.3146	902524	367554	43
Bb16-135	M	NWON	Big Island	49.1215	-94.3146	910205	372246	530*
Bb16-136	M	NWON	Big Island	49.1215	-94.3146	903319	391410	227
Bb16-138	M	NWON	Big Island	49.1215	-94.3146	902392	366459	43
Bb16-139	M	NWON	Big Island	49.1215	-94.3146	902881	393673	221
Bb16-33.3	F	NWON	Birch Island	49.6322	-94.5762	902806	348146	302
Bb16-132	M	NWON	Manitou Rapids	48.6345	-93.9203	903184	392085	221
Bb16-47	F	NWON	Manitou Rapids	48.6345	-93.9203	909965	402625	30
Bb16-198	F	NS	Bedford	44.7186	-63.6526	903037	432289	3
Bb16-202	F	NS	Bedford	44.7186	-63.6526	902430	440730	16
Bb16-150	M	NS	Lunenburg	44.3766	-64.2573	902272	421805	19

Bb16-170	M	NS	Lunenburg	44.3766	-64.2573	903062	425860	3
Bb16-174	M	NS	Lunenburg	44.3766	-64.2573	910083	347266	37
Bb16-175	M	NS	Lunenburg	44.3766	-64.2573	903144	424359	3
Bb16-178-1	M	NS	Lunenburg	44.3766	-64.2573	902948	434013	3
Bb16-178-2	M	NS	Lunenburg	44.3766	-64.2573	903046	369860	12
Bb16-268	F	NS	Lunenburg	44.3766	-64.2573	909876	335250	14
Bb16-181	F	NS	Pictou	45.6454	-62.5503	902480	403425	19
Bb16-182	F	NS	Pictou	45.6454	-62.5503	909674	420102	3
Bb16-183	F	NS	Pictou	45.6454	-62.5503	902934	398424	4
Bb16-184	F	NS	Pictou	45.6454	-62.5503	903198	438104	3
Bb16-186	F	NS	Pictou	45.6454	-62.5503	902759	396778	3
Bb16-188	F	NS	Pictou	45.6454	-62.5503	902869	413970	4
Bb16-193-2	M	NS	Pictou	45.6454	-62.5503	902543	444867	16
Bb16-142	F	NS	Shelburne	43.6712	-65.3420	907013	405971	3
Bb16-145	F	NS	Shelburne	43.6712	-65.3420	902450	417281	19
Bb16-146	F	NS	Shelburne	43.6712	-65.3420	902999	405809	3
Bb16-149	F	NS	Shelburne	43.6712	-65.3420	903159	426922	3
Bb16-163	F	NS	Shelburne	43.6712	-65.3420	910351	475741	1
Bb16-164	F	NS	Shelburne	43.6712	-65.3420	899509	464427	16
Bb16-167	F	NS	Shelburne	43.6712	-65.3420	903104	406842	12
Bb16-249	F	NS	Shelburne	43.6712	-65.3420	906016	433269	4
Bb16-250	F	NS	Shelburne	43.6712	-65.3420	902872	392030	12

Table S2. Additional sequences included in phylogenetic analysis.

Sequence	Accession Number	Location	MLST ST
CA382	CP005925.1	California,US	ST2
CA8	ADMY01000001.1-ADMY01000007.1	California,US	ST742
PAbe	SRR1737741	Munich	ST1
PAli	SRR1739917	Regensburg	ST1
JD1	CP002312.1	Massachusetts	ST11
29805	ABJX02000001.1-ABJX02000038.1	Connecticut,US	ST12
72a	ABGJ02000001.1-ABGJ02000006.1	NewYork,US	ST14
94a	ABGK02000001.1-ABGK02000009.1	NewYork,US	ST18
N40	CP002228.1	NewYork,US	ST19
PBre	SRR1739918	Munich	ST20
PKu	SRR1739934	Bad Mergentheim	ST20
PMeh	SRR1739936	Garmisch	ST20
ZS7	CP001205.1	Germany,EU	ST20
PFhe_I	SRR1739920	Munich	ST21
PFhe_II	SRR1739921	Munich	ST21
PGl	SRR1739923	Bayreuth	ST21
PSst	SRR1739938	Tübingen	ST21
WI91-23	ABJW02000001.1-ABJW02000031.1	Wisconsin,US	ST228
PDri	SRR1739919	Munich	ST24
PKif_I	SRR1739925	Munich	ST24
PKif_II	SRR1739926	Munich	ST24
Z41293	SRR1739939	Germany	ST27
PFi_I	SRR1739922	Munich	ST284
PLue	SRR1739935	Munich	ST3
Bol26	ABCW02000001.1-ABCW02000005.1	Italy,EU	ST332
CA-11.2A	ABJY02000001.1-ABJY02000014.1	California,US	ST333
118a	ABGI02000001.1-ABGI02000008.1	NewYork,US	ST34
156a	ABCV02000001.1	NewYork,US	ST4
SV1	ABJZ02000001.1-ABJZ02000005.1	Finland,EU	ST414
64b	ABKA02000001.1-ABKA02000006.1	NewYork,US	ST59
B31	NC_001318.1	NewYork,US	ST1
B. bissettiae	CP002746	California, US	
B31 plasmid cp26	NC_001903.1		
B31 plasmid cp32-1	NC_000948.1		
B31 plasmid cp32-3	NC_000949.1		
B31 plasmid cp32-4	NC_000950.1		
B31 plasmid cp32-6	NC_000951.1		
B31 plasmid cp32-7	NC_000952.1		
B31 plasmid cp32-8	NC_000953.1		
B31 plasmid cp32-9	NC_000954.1		
B31 plasmid cp9	NC_001904.1		
B31 plasmid lp17	NC_001849.2		
B31 plasmid lp21	NC_000955.2		

B31 plasmid lp25	NC_001850.1
B31 plasmid lp28-1	NC_001851.2
B31 plasmid lp28-2	NC_001852.1
B31 plasmid lp28-3	NC_001853.1
B31 plasmid lp28-4	NC_001854.1
B31 plasmid lp36	NC_001855.1
B31 plasmid lp38	NC_001856.1
B31 plasmid lp5	NC_000957.1
B31 plasmid lp54	NC_001857.2
B31 plasmid lp56	NC_000956.1

Table S3. The proteins that are known or likely to be surface-exposed, or to be proteins of the western blot diagnostic algorithm for Lyme disease, used in the gene selection analysis.

NCBI protein_id	CDS	Gene	Locus_Tag	Product
AAB91505.2	bmpD CDS	bmpD	BB_0385	basic membrane protein D (bmpD)
AAC66397.2	membrane protein CDS		BB_0006	membrane protein
AAC66426.1	outer membrane protein P13 CDS		BB_0034	outer membrane protein P13
AAC66428.1	lipoprotein, putative CDS		BB_0028	lipoprotein, putative
AAC66436.2	P115 protein CDS		BB_0045	P115 protein
AAC66457.2	putative protein-export membrane protein SecG CDS		BB_0054	putative protein-export membrane protein SecG
AAC66467.1	membrane protein, putative CDS		BB_0072	membrane protein, putative
AAC66497.1	basic membrane protein CDS		BB_0108	basic membrane protein
AAC66515.1	membrane protein CDS		BB_0117	membrane protein
AAC66527.2	membrane fusion protein CDS		BB_0141	membrane fusion protein
AAC66532.2	outer membrane efflux protein CDS		BB_0142	outer membrane efflux protein
AAC66541.1	p41 CDS		BB_0147	flagellin subunit B
AAC66544.1	lipoprotein, putative CDS		BB_0155	lipoprotein, putative
AAC66550.1	S2 lipoprotein CDS		BB_0158	S2 lipoprotein
AAC66563.2	outer membrane protein CDS		BB_0167	outer membrane protein
AAC66581.1	lipoprotein, putative CDS		BB_0193	lipoprotein, putative
AAC66603.2	flagellar motor switch protein CDS		BB_0221	flagellar motor switch protein
AAC66613.1	lipoprotein, putative CDS		BB_0213	lipoprotein, putative
AAC66654.1	flgB CDS	flgB	BB_0294	flagellar basal-body rod protein FlgB
AAC66655.1	flgC CDS	flgC	BB_0293	flagellar basal-body rod protein FlgC
AAC66656.1	fliE CDS	fliE	BB_0292	flagellar hook-basal body complex protein (FliE)
AAC66657.1	fliF CDS	fliF	BB_0291	flagellar M-ring protein FliF
AAC66658.1	fliG CDS	fliG	BB_0290	flagellar motor switch protein FliG
AAC66659.1	flagellar assembly protein FliH CDS		BB_0289	flagellar assembly protein FliH
AAC66660.1	fliI CDS	fliI	BB_0288	flagellar protein export ATPase FliI
AAC66661.1	flagellar protein CDS		BB_0287	flagellar protein
AAC66662.2	flagellar protein CDS		BB_0286	flagellar protein
AAC66663.2	flagellar protein CDS		BB_0285	flagellar protein
AAC66664.1	flgD CDS	flgD	BB_0284	flagellar hook capping protein
AAC66665.1	flagellar hook protein FlgE CDS		BB_0283	flagellar hook protein FlgE
AAC66668.1	flagellar motor apparatus CDS		BB_0280	flagellar motor apparatus
AAC66669.1	fliL CDS	fliL	BB_0279	flagellar basal body-associated protein FliL
AAC66670.1	fliM CDS	fliM	BB_0278	flagellar motor switch protein FliM
AAC66671.1	flagellar switch protein FliY CDS		BB_0277	flagellar switch protein FliY
AAC66672.1	flagellar protein FliZ CDS		BB_0276	flagellar protein FliZ
AAC66673.1	fliP CDS	fliP	BB_0275	flagellar biosynthetic protein FliP

AAC66674.1	fliQ CDS	fliQ	BB_0274	flagellar biosynthetic protein FliQ
AAC66675.2	fliR CDS	fliR	BB_0273	flagellar biosynthetic protein FliR
AAC66676.1	flhB CDS	flhB	BB_0272	flagellar biosynthetic protein FlhB
AAC66677.1	flhA CDS	flhA	BB_0271	flagellar biosynthesis protein FlhA
AAC66678.1	flagellar biosynthesis protein FlhF (Flagella-associated GTP-bindingprotein) CDS		BB_0270	flagellar biosynthesis protein FlhF (Flagella-associated GTP-bindingprotein)
AAC66683.2	heat shock protein 70 CDS		BB_0264	heat shock protein 70
AAC66706.1	bacterial extracellular solute-binding protein, family 5 CDS		BB_0330	bacterial extracellular solute-binding protein, family 5
AAC66708.2	bacterial extracellular solute-binding protein, family 5 CDS		BB_0328	bacterial extracellular solute-binding protein, family 5
AAC66738.1	membrane protein, putative CDS		BB_0353	membrane protein, putative
AAC66748.1	la7 CDS	la7	BB_0365	outer surface 22 kda lipoprotein (antigen IpLA7)
AAC66753.1	P31-23 protein CDS		BB_0373	P31-23 protein
AAC66756.1	bmpC CDS	bmpC	BB_0384	basic membrane protein C (bmpC)
AAC66757.1	bmpA CDS	bmpA	BB_0383	basic membrane protein A (bmpA), immunodominant antigen P39
AAC66758.1	bmpB CDS	bmpB	BB_0382	basic membrane protein B (bmpB)
AAC66778.1	lipoprotein, putative CDS		BB_0398	lipoprotein, putative
AAC66791.1	membrane protein, putative CDS		BB_0412	membrane protein, putative
AAC66868.2	lipoprotein, putative CDS		BB_0475	lipoprotein, putative
AAC66898.1	membrane protein, putative CDS		BB_0539	membrane protein, putative
AAC66908.1	fliS CDS	fliS	BB_0550	flagellar protein FliS
AAC66949.1	p66 CDS	p66	BB_0603	integral outer membrane protein P66
AAC66985.1	lipoprotein, putative CDS		BB_0628	lipoprotein, putative
AAC66991.1	heat shock protein CDS		BB_0655	heat shock protein
AAC66992.1	secF CDS	secF	BB_0653	protein-export membrane protein SecF
AAC67012.2	lipoprotein, putative CDS		BB_0664	lipoprotein, putative
AAC67025.2	flagellar filament outer layer protein CDS		BB_0668	flagellar filament outer layer protein
AAC67027.1	membrane protein, putative CDS		BB_0674	membrane protein, putative
AAC67038.1	lipoprotein, putative CDS		BB_0689	lipoprotein, putative
AAC67090.1	p83/100 CDS	p83/100	BB_0744	Borrelia P83/P100 antigen
AAC67102.1	membrane protein, putative CDS		BB_0759	membrane protein, putative
AAC67131.2	flagellar hook-basal body complex protein CDS		BB_0775	flagellar hook-basal body complex protein
AAC67132.1	flgG CDS	flgG	BB_0774	flagellar basal-body rod protein FlgG
AAC67133.1	flagellar P-ring protein (Basal body P-ring protein) CDS		BB_0772	flagellar P-ring protein (Basal body P-ring protein)
AAC67139.1	outer membrane protein CDS		BB_0795	outer membrane protein
AAC67146.2	mviN CDS	mviN	BB_0810	integral membrane protein MviN

AAC67160.1	lipoprotein, putative CDS	BB_0806	lipoprotein, putative
AAC67172.1	lipoprotein, putative CDS	BB_0823	lipoprotein, putative
AAC67183.1	lipoprotein, putative CDS	BB_0832	lipoprotein, putative
AAC67186.1	lipoprotein, putative CDS	BB_0840	lipoprotein, putative

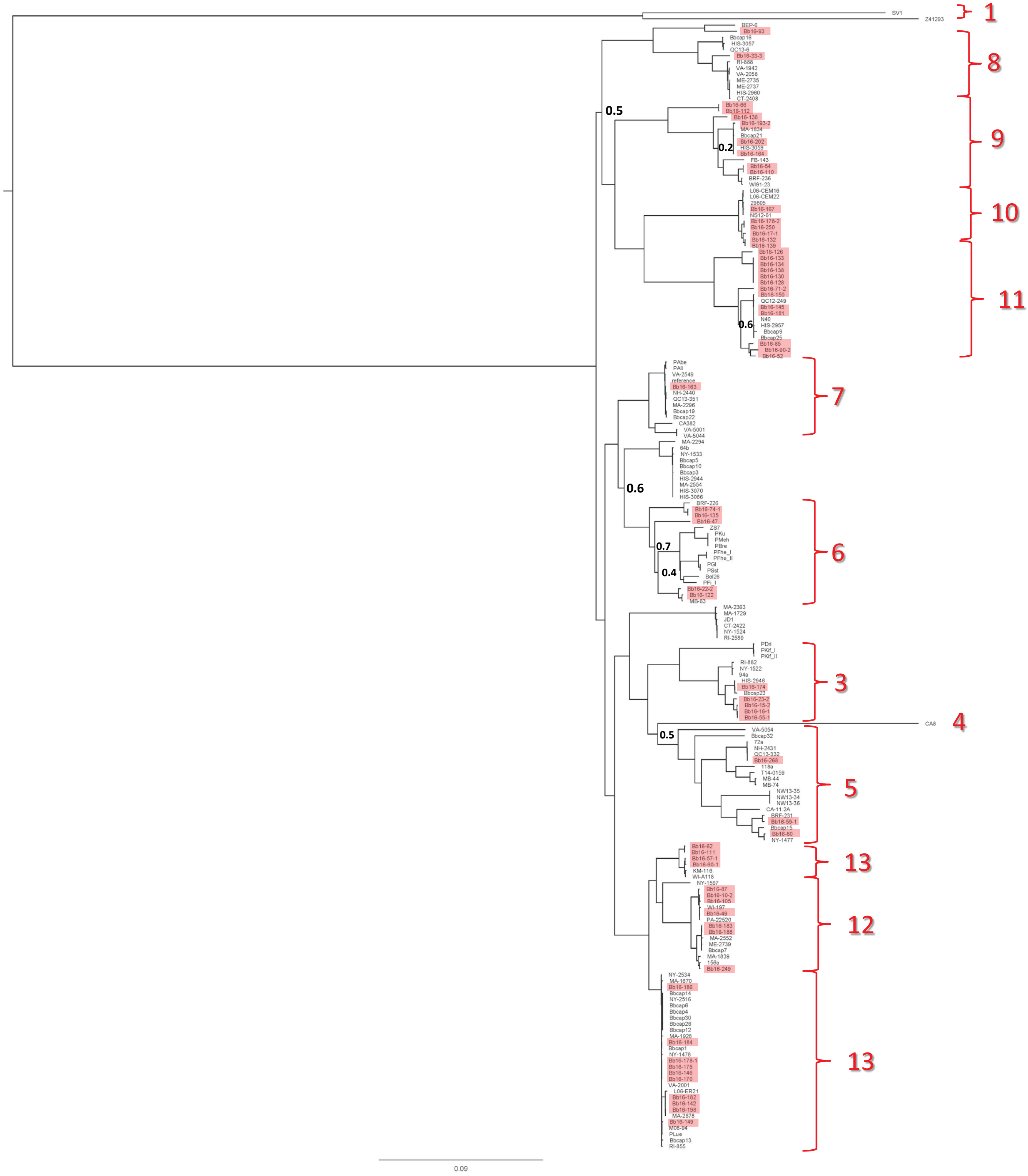

Fig S1. The midpoint rooted phylogenetic tree of the *B. burgdorferi* ss chromosome generated via the SNVPhyl pipeline using all sequences obtained in this study (highlighted in red), currently available published full sequences from samples collected elsewhere in North America and Europe, as well as selected sequences from North America obtained by Walter et al.[S1]. The brackets and numbers in red correspond to the numbering of major clades in the whole chromosome tree in Fig 6 in the main document. The clades remain unchanged except that clade 12 separates the well supported subclades of clade 13 in Fig 5. Likelihood values for branches with a value less than 0.9 are shown.

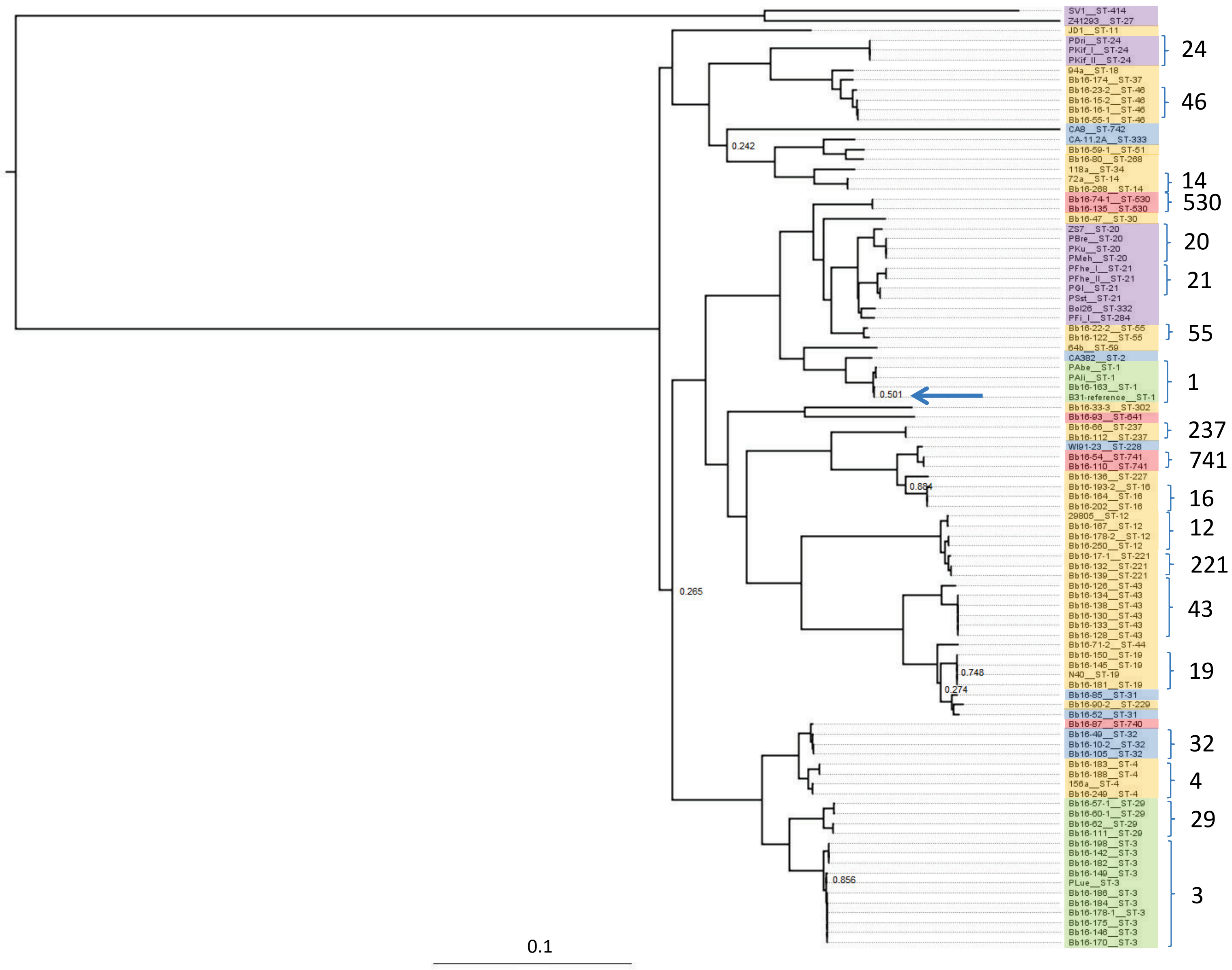

Fig S2. The midpoint rooted phylogenetic tree of the *B. burgdorferi* ss chromosome generated via the SNVPhyl pipeline using all sequences obtained in this study and currently available published full sequences from samples collected elsewhere in North America and Europe as in Fig 2 in the main text. The clustering of strains carrying the same MLST ST is identified by brackets with the ST numbers to the right. The blue arrow indicates the reference strain B31, and the colour coding indicates the geographic occurrence of the MLST types of the sequences: blue = USA only; red = Canada only, yellow = USA and Canada; violet = Europe; green = North America and Europe.

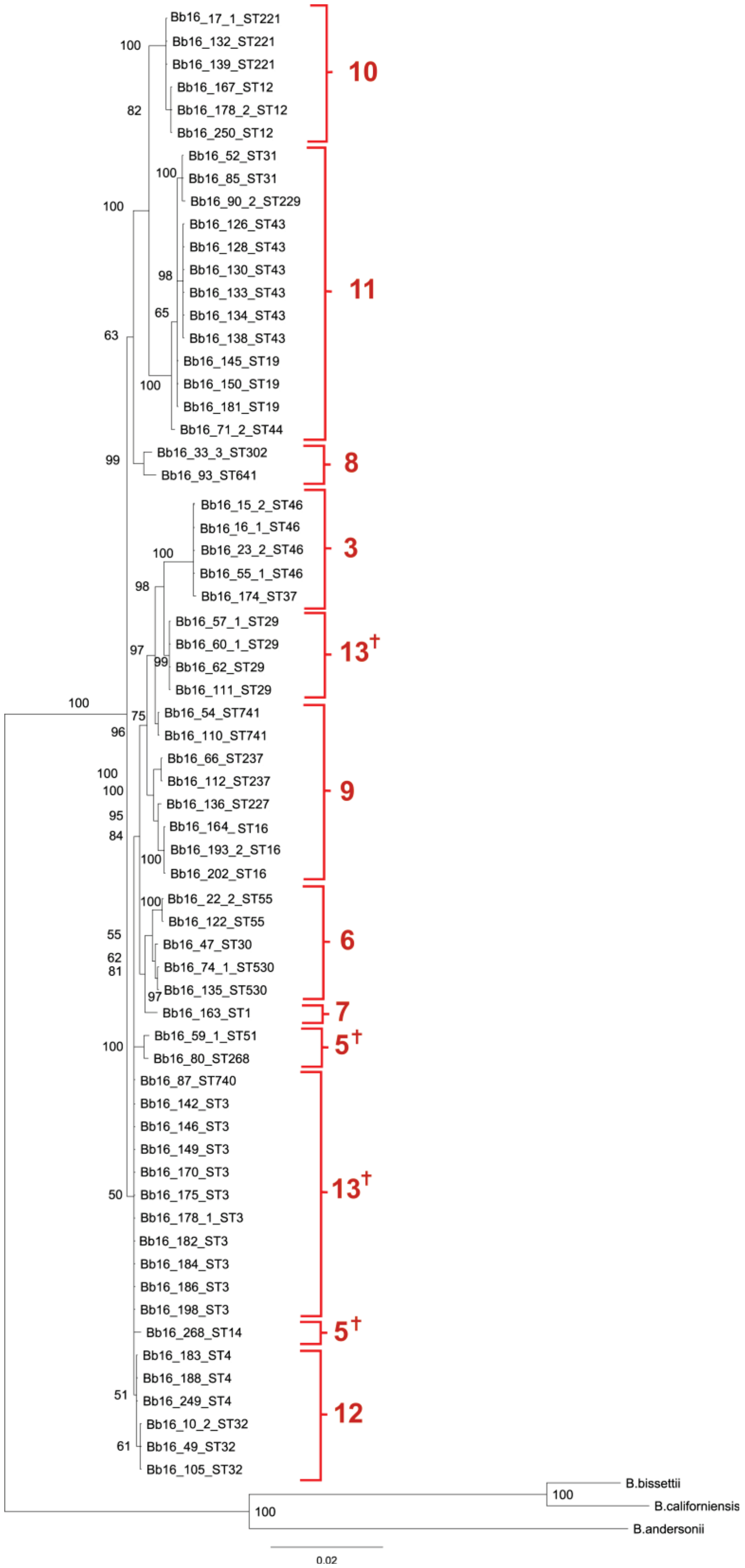

Fig S3. A Bayesian phylogenetic tree based on the MLST sequences of the strains. The brackets and numbers in red correspond to the numbering of major clades in the whole chromosome tree in Fig 6 in the main document. Major clades in the whole chromosome and MLST trees were the same with the exception of clade 13 which occurred as two clades of the MLST tree, one of which separated asterisked clade 5 into two. However the separation of clade 13 in the MLST tree occurred according to minor clades of clade 13 evident in the whole chromosome tree. The differences between the MLST and whole chromosome trees are indicated by the symbol †.

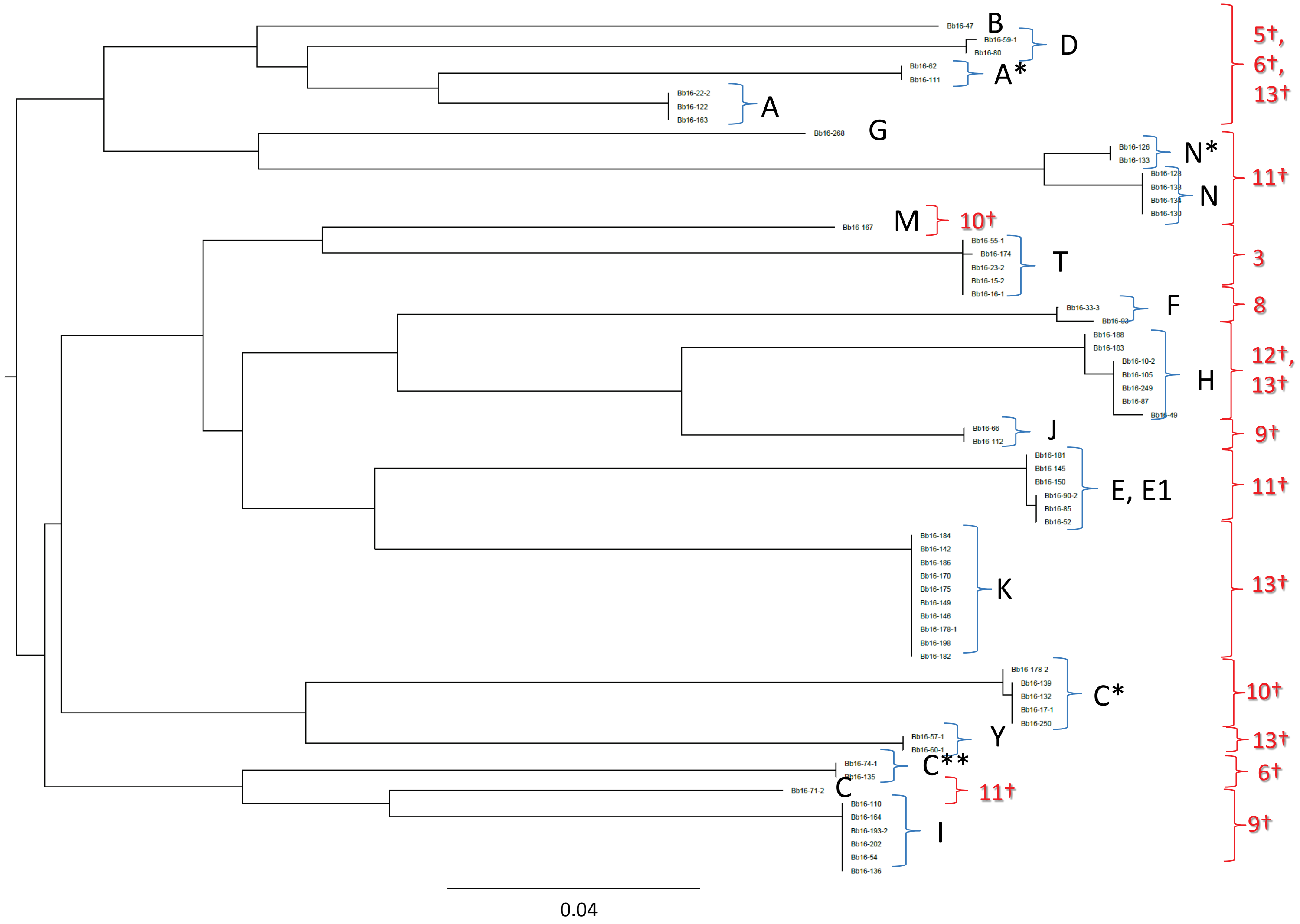

Fig S4. A Bayesian phylogenetic tree based on the *ospC* sequences of the strains. Letters indicate the *ospC* major groups of the sequences. Letters with asterisks indicate novel *ospC* major groups as described in the results section of the main document. The brackets and numbers in red correspond to the numbering of major clades in the whole chromosome tree in Fig 6 in the main document. Major clades of the *ospC* tree that were different to the whole chromosome tree (by being split or by including sequences from other clades of the whole chromosome tree) are indicated by the symbol †.

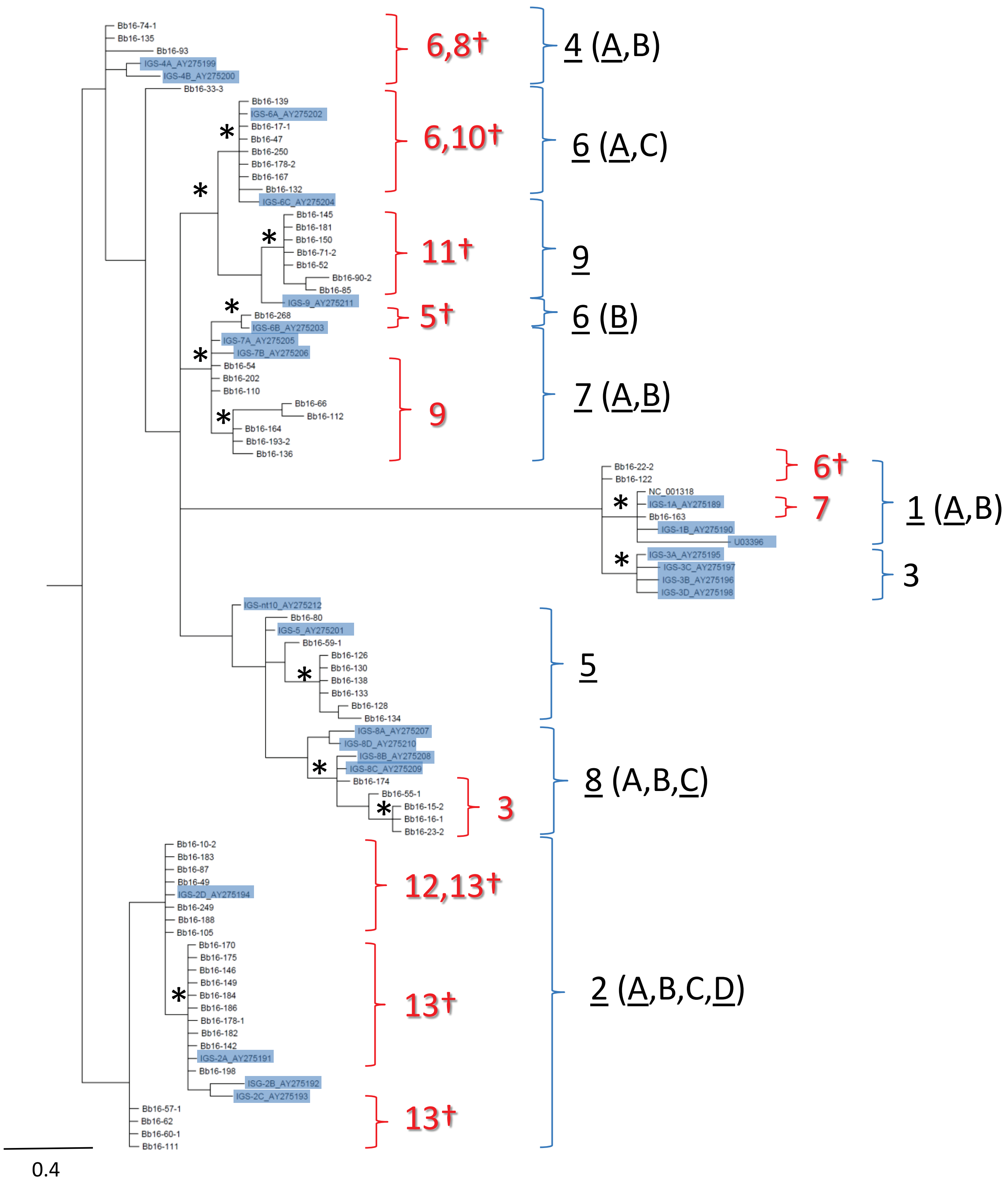

Fig S5. A Bayesian phylogenetic tree based on the IGS sequences of the strains as well as reference sequences from Genbank (highlighted in blue). Brackets and numbers in red immediately to the right of the strain IDs correspond to the numbering of major clades in the whole chromosome tree in Fig 6 in the main document. Major clades of the IGS tree that were different to the whole chromosome tree (by being split or by including sequences from other clades of the whole chromosome tree) are indicated by the symbol †. Blue brackets to the right indicate the IGS genotypes (numbers) and sub-genotypes (letters). Underlined IGS numbers and letters indicate that the Canadian strains were of these IGS types. Asterisks indicate nodes for which posterior probabilities are < 90%.

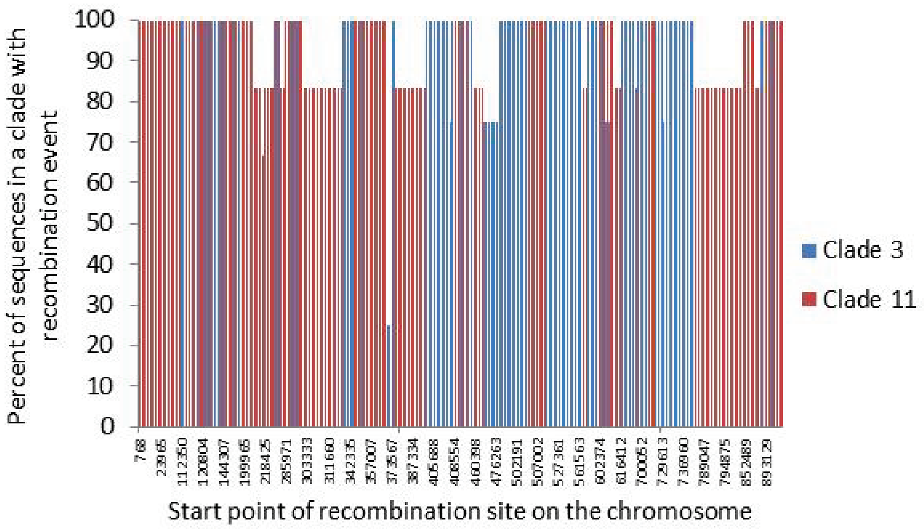

Fig S6. Inter-clade variation in regions of recombination illustrated by comparisons between two clades. The chart shows the percentage of strains of clades 3 and 11 of the phylogenetic tree (see Figs 4 and 6 in the main manuscript) that had evidence of recombination within each of the 247 locations on the chromosome where recombination was detected. The start point of the region of recombination on the chromosome is identified on the x-axis. Possible recombination was detected in 127 locations for strains of clade 3, and 162 locations for strains of clade 11. However at only 42 locations on the chromosome were possible recombination events detected in both clades.

Selection of sequences from Walter et al.[S1] for inclusion in phylogenetic analysis

Of the 146 sequences reported in Walter et al.[S1] 8 were excluded from consideration as they did not meet our minimum genome coverage requirements of 10x across 80% of the genome when compared to the B31 reference sequence (Table S4). In order to assess the "strain purity" of the remaining samples the sequencing reads were mapped to the B31 genome sequence using Bowtie2 and variants were called using a simple diploid model with FreeBayes[S2] with settings of minimum mapping quality = 30, minimum base quality = 20, minimum supporting allele qsum = 0, genotype variant threshold = 0 and minimum coverage = 10. Sites were only considered polymorphic if the allele balance fell between 0.25 and 0.75 as these were considered the most likely to give rise to chimeric sequences. Allele frequencies outside of this range were assumed to result in a single sequence representative of the majority population. This produced an additional criterion for including strains in phylogenetic analysis (Table S5), and excluding them (Table S6). As a basis of comparison this analysis was also applied to the sequence data for strains derived from our single colonies, and previously published sequences (Table S6).

All 146 strains were also run through SRST2 using the *B. burgdorferi* MLST typing scheme in order to see if ST's could be assigned. An "*" denotes a minor variation (typically SNPs) from a known allele/ST and may potentially represent a new allele and sequence type. A "?" indicates uncertainty in the allele called typically due to coverage issues. A "NF" in the ST designation indicates the allele combination was not found but otherwise consists of known alleles and likely represents a new ST.

References:

S1. Walter, K. S., Carpi, G., Caccone, A. & Diuk-Wasser, M. A. Genomic insights into the ancient spread of Lyme disease across North America. *Nat. Ecol. Evol.* **1**, 1569-1576 (2017).

S2. Garrison, E. & Marth G. Haplotype-based variant detection from short-read sequencing. rXiv:1207.3907 [q-bio.GN] https://arxiv.org/abs/1207.3907 (2012).

Table S4. Strains excluded due to insufficient genome coverage below 80% of the genome covered at a minimum of 10X.

Sample	Genome Coverage @ 10x	ST	clpA	clpX	nifS	pepX	pyrG	recG	rplB	uvrA	mismatches	uncertainty	depth
BEP-11	70.40%	56*?	24?	14	4*?	18?	11?	19	1	12?	nifS_4/12holes	clpA_24/edge1.0;nifS_4/edge0.0;pepX_18/edge2.0;pyrG_11/edge1.5;uvrA_12/edge2.0	12.3
LP-79	78.02%	NF*?	6*	12?	1*	2?	2?	15	1	2	clpA_6/1snp;nifS_1/2snp	clpX_12/edge1.0;pepX_2/edge2.0;pyrG_2/edge1.0	16.4
MI11-837	0.11%	-	-	-	-	-	-	-	-	-			
MI-5195	16.00%	NF*?	5*?	5	12?	8*?	3*?	3*?	94*?	14*?	clpA_5/1snp13holes;pepX_8/1snp3holes;pyrG_3/1snp;recG_3/22holes;rplB_94/16holes;uvrA_14/2snp22holes	clpA_5/edge0.0;nifS_12/edge1.0;pepX_8/edge0.0;pyrG_3/edge2.0;recG_3/edge0.0;rplB_94/edge0.0;uvrA_14/edge0.0	9.8
NY-2396	56.90%	3*?	4*?	1	1*?	1	1*	6	1	7?	clpA_4/11holes;nifS_1/2holes;pyrG_1/10holes	clpA_4/edge0.0;nifS_1/edge0.0;uvrA_7/edge2.0	11.2
NY-2401	0.32%	NF*?	-	13*?	1?	-	-	-	-	7*?	clpX_13/25holes;uvrA_7/47holes	clpX_13/edge0.0;nifS_1/edge1.0;uvrA_7/edge0.0	3.1
QC12-207	65.69%	3*?	4	1?	1?	1?	1	6	1*?	7?	rplB_1/18holes	clpX_1/edge1.0;nifS_1/edge1.0;pepX_1/edge2.0;rplB_1/edge0.0;uvrA_7/edge2.0	11.9
UR14-2158	68.10%	NF*?	8*?	14	6*	6?	5*?	6?	1	7	clpA_8/5holes;nifS_6/1snp;pyrG_5/5holes	clpA_8/edge0.0;pepX_6/edge2.0;pyrG_5/edge0.0;recG_6/edge2.0	14.2

Table S5. Strains included in the phylogenetic analysis.

Sample	Poly-morphic	Mono-morphic	%Poly	ST	clpA	clpX	nifS	pepX	pyrG	recG	rplB	uvrA	mismatches	uncertainty	depth
BRF-236	0	3581	0.00	228	12	2	1	2	1	16	2	2	0	-	55.6
NH-2431	0	3317	0.00	14	9	1	1	7	1	6	1	10	0	-	33.7
NH-2440	0	37	0.00	1	1	1	1	1	1	1	1	1	0	-	121.5
MB-74	1	3490	0.03	301	12	1	1	7	112	6	1	10	0	-	47.8
Bbcap15	3	3362	0.09	226	8	1	15	94	2	20	1	7	0	-	48.3
MB-44	4	3697	0.11	301	12	1	1	7	112	6	1	10	0	-	118.2
RI-888	4	3415	0.12	9	10	5	4	6	1	6	1	6	0	-	69.4
VA-1942	5	3678	0.14	36	10	5	4	6	1	15	1	6	0	-	249.8
Bbcap9	6	3971	0.15	19	4	4	3	3	3	3	3	3	0	-	250.5

Bbcap21	6	3695	0.16	16	2	2	1	2	2	2	2	2	0	-	131.9
RI-882	6	3171	0.19	18	7	6	6	1	1	5	5	5	0	-	28.4
Bbcap16	7	3464	0.20	8	5	5	4	5	5	5	1	6	0	-	113.4
RI-855	7	3439	0.20	3	4	1	1	1	1	6	1	7	0	-	115.9
VA-5001	2	932	0.21	403*	1	1	1	1	1	1*	1	11	recG_1/1snp	-	63.1
NY-1597	7	3198	0.22	NF	158	1	1	1	1	19	1	7	0	-	40.1
Bbcap25	8	3582	0.22	19	4	4	3	3	3	3	3	3	0	-	47.1
NW13-34	9	3698	0.24	225	8	2	5	93	2	8	1	84	0	-	537.6
Bbcap10	5	2053	0.24	7	6	1	5	1	1	7	1	8	0	-	494.7
KM-116	8	3154	0.25	NF	18	12	1	11	2	15	1	7	0	-	26.3
MA-1928	9	3472	0.26	3	4	1	1	1	1	6	1	7	0	-	137.3
NY-2516	9	3456	0.26	3	4	1	1	1	1	6	1	7	0	-	187.8
MA-2294	5	1917	0.26	NF*	6	1	5	1	1*	6*	1	7*	pyrG_1/1snp;recG_6/1snp;uvrA_7/1snp	-	46.2
NY-1477	9	3394	0.26	268	8	1	15	94	2	20	96	7	0	-	55.5
Bbcap23	10	3741	0.27	37	7	6	12	1	1	5	5	5	0	-	151.5
NW13-36	10	3622	0.28	225	8	2	5	93	2	8	1	84	0	-	223.6
MA-1670	9	3201	0.28	3	4	1	1	1	1	6	1	7	0	-	47.4
HIS-2946	11	3873	0.28	37	7	6	12	1	1	5	5	5	0	-	667.9
QC13-332	11	3790	0.29	14	9	1	1	7	1	6	1	10	0	-	387.5
ME-2735	11	3686	0.30	36	10	5	4	6	1	15	1	6	0	-	421.4
VA-5054	12	3881	0.31	NF*	21*	1	12	8	168*	3*	1	14*	clpA_21/1snp;pyrG_168/1indel;recG_3/1snp;uvrA_14/1snp1indel	-	961.6
Bbcap13	11	3553	0.31	3	4	1	1	1	1	6	1	7	0	-	540.1
ME-2737	11	3476	0.32	36	10	5	4	6	1	15	1	6	0	-	100.3
Bbcap32	12	3673	0.33	40	22	1	5	8	1	18	11	10	0	-	144.2
NY-2534	13	3551	0.36	3	4	1	1	1	1	6	1	7	0	-	694.5
Bbcap14	13	3430	0.38	3	4	1	1	1	1	6	1	7	0	-	135.7
HIS-3059	14	3634	0.38	16	2	2	1	2	2	2	2	2	0	-	89.5
NY-1478	14	3555	0.39	3	4	1	1	1	1	6	1	7	0	-	741.2
Bbcap6	14	3533	0.39	3	4	1	1	1	1	6	1	7	0	-	559.2
Bbcap26	14	3300	0.42	3	4	1	1	1	1	6	1	7	0	-	66.0
L06-CEM16	16	3669	0.43	12	3	3	2	4	3	4	4	4	0	-	73.6
HIS-3070	9	2054	0.44	7	6	1	5	1	1	7	1	8	0	-	593.7
VA-2058	15	3399	0.44	36	10	5	4	6	1	15	1	6	0	-	70.0
QC13-6	15	3321	0.45	8	5	5	4	5	5	5	1	6	0	-	76.7
Bbcap4	17	3536	0.48	3	4	1	1	1	1	6	1	7	0	-	537.9
L06-CEM22	17	3474	0.49	12	3	3	2	4	3	4	4	4	0	-	45.7

HIS-2957	19	3877	0.49	19	4	4	3	3	3	3	3	3	0	-	127.5
HIS-3066	10	2036	0.49	7	6	1	5	1	1	7	1	8	0	-	213.1
Bbcap7	16	3252	0.49	4	8	1	1	1	4	6	1	7	0	-	68.9
HIS-2944	10	2031	0.49	7	6	1	5	1	1	7	1	8	0	-	231.2
T14-0159	19	3805	0.50	52	12	1	1	7	1	6	1	10	0	-	800.3
VA-5044	5	965	0.52	403*	1	1	1	1	1	1*	1	11	recG_1/1snp	-	599.3
FB-143	22	3950	0.55	NF	12	2	2	2	1	8	1	2	0	-	884.9
WI-A118	25	3469	0.72	29	18	12	1	11	2	15	1	2	0	-	142.6
MA-1729	28	3377	0.82	11	5	7	5	1	6	1	4	9	0	-	151.3
NW13-35	32	3639	0.87	225	8	2	5	93	2	8	1	84	0	-	379.0
MA-1839	32	3546	0.89	4	8	1	1	1	4	6	1	7	0	-	370.0
VA-2001	41	3526	1.15	3	4	1	1	1	1	6	1	7	0	-	572.2
RI-2589	38	3203	1.17	11	5	7	5	1	6	1	4	9	0	-	47.9
Bbcap1	29	2338	1.23	3?	4?	1	1	1	1	6	1	7	0	clpA_4/edge2.0	18.5
CT-2422	47	3456	1.34	11	5	7	5	1	6	1	4	9	0	-	306.9
PA-22520	43	3103	1.37	32	8	1	1	1	4	16	1	7	0	-	49.8
MA-2363	48	3370	1.40	11	5	7	5	1	6	1	4	9	0	-	140.1
ME-2739	56	3451	1.60	4	8	1	1	1	4	6	1	7	0	-	553.2
Bbcap30	60	3478	1.70	3	4	1	1	1	1	6	1	7	0	-	768.0
MA-2678	62	3480	1.75	3	4	1	1	1	1	6	1	7	0	-	598.2
BRF-231	66	3657	1.77	NF*	8	1	15	15*	12	20	1	7	pepX_15/2snp	-	601.4
HIS-2960	67	3129	2.10	36	10	5	4	6	1	15	1	6	0	-	43.3
BEP-6	82	3686	2.18	56	24	14	4	18	11	19	1	12	0	-	269.2
HIS-3057	75	3304	2.22	8	5	5	4	5	5	5	1	6	0	-	139.8
L06-ER21	63	2718	2.27	3	4	1	1	1	1	6	1	7	0	-	30.6
NY-1533	44	1759	2.44	7	6	1	5	1	1	7	1	8	0	-	37.2
CT-2408	101	3495	2.81	36	10	5	4	6	1	15	1	6	0	-	254.1
MA-1834	114	3694	2.99	16	2	2	1	2	2	2	2	2	0	-	750.8
MA-2554	53	1697	3.03	7	6	1	5	1	1	7	1	8	0	-	39.1
QC12-249	130	3880	3.24	19	4	4	3	3	3	3	3	3	0	-	674.4
WI-197	119	2915	3.92	32	8	1	1	1	4	16	1	7	0	-	75.0
Bbcap3	65	1588	3.93	7	6	1	5	1	1	7	1	8	0	-	89.7
NY-1522	148	3558	3.99	18	7	6	6	1	1	5	5	5	0	-	353.9
Bbcap22	2	47	4.08	1	1	1	1	1	1	1	1	1	0	-	794.4
NY-1524	140	2960	4.52	11	5	7	5	1	6	1	4	9	0	-	114.4
MA-2552	154	3247	4.53	4	8	1	1	1	4	6	1	7	0	-	646.0

NS12-61	181	3460	4.97	12	3	3	2	4	3	4	4	4	0	-	673.5
MA-2296	2	31	6.06	1	1	1	1	1	1	1	1	1	0	-	212.2
M08-94	195	3005	6.09	3?	4	1	1	1?	1	6	1	7	0	pepX_1/edge1.0	108.4
VA-2549	1	15	6.25	1	1	1	1	1	1	1	1	1	0	-	341.5
QC13-351	3	37	7.50	1	1	1	1	1	1	1	1	1	0	-	277.0
MB-63	163	1901	7.90	NF*	23	1	17	20	2	191*	160	10	recG_191/61holes	-	339.9
Bbcap12	218	2449	8.17	3	4	1	1	1	1	6	1	7	0	-	193.6
Bbcap19	4	42	8.70	1*	1*	1	1	1	1	1	1	1	clpA_1/1snp	-	30.3
Bbcap5	135	1340	9.15	7	6	1	5	1	1	7	1	8	0	-	189.1
BRF-226	174	1627	9.66	530	14	1	5	2	2	1	1	10	0	-	631.0

Table S6. Strains excluded from the phylogenetic analysis due to greater than 10% polymorphic sites

Sample	Poly-morphic	Mono-morphic	%Poly	ST	clpA	clpX	nifS	pepX	pyrG	recG	rplB	uvrA	mismatches	uncertainty	depth
T13-2047	273	1527	15.17	55	23	1	17	20	2	1	1	10	0	-	119.8
MA-1720	588	2933	16.70	40	22	1	5	8	1	18	11	10	0	-	449.8
Bbcap24	354	1409	20.08	7	6	1	5	1	1	7	1	8	0	-	66.6
Bbcap2	421	1636	20.47	7	6	1	5	1	1	7	1	8	0	-	362.9
NY-1556	8	31	20.51	1?	1?	1	1	1	1	1	1	1	0	clpA_1/edge2.0	24.6
NY-2455	672	2541	20.92	18	7	6	6	1	1	5	5	5	0	-	54.6
M08-56	781	2805	21.78	16*	2*	2	1	2	2	2	2	2	clpA_2/1snp	-	44.6
HIS-2939	767	2728	21.95	16	2	2	1	2	2	2	2	2	0	-	596.0
Bbcap28	603	2116	22.18	18	7	6	6	1	1	5	5	5	0	-	136.3
NY-2464	648	2216	22.63	12*	3	3	2	4	3	4	4	4*	uvrA_4/1snp	-	114.4
NY-2387	935	2635	26.19	12	3	3	2	4	3	4	4	4	0	-	46.2
HIS-3047	661	1474	30.96	4	8	1	1	1	4	6	1	7	0	-	565.7
FB-153	1086	2035	34.80	29	18	12	1	11	2	15	1	2	0	-	586.9
Bbcap27	1031	1601	39.17	3	4	1	1	1	1	6	1	7	0	-	71.3
FB-141	1390	2045	40.47	397	15	121	12	138	1	135	1	123	0	-	21.6
Bbcap20	598	860	41.02	14	9	1	1	7	1	6	1	10	0	-	118.8
NY-2523	1877	2293	45.01	19	4	4	3	3	3	3	3	3	0	-	801.0
BEP-10	3535	4178	45.83	NF*	5	1	1	6	11*	6	1	10*	pyrG_11/2snp;uvrA_10/1snp	-	670.0
FB-158	2981	3489	46.07	NF*	11	12	12	94	2	15	1	123*	uvrA_123/3snp	-	50.1
MB-48	3255	3758	46.41	NF*	107*	2*	1	116	1*	6	76*	2*	clpA_107/4snp;clpX_2/1snp;pyrG_1/2snp;rplB_76/3snp;uvrA_2/2snp	-	857.7

MA-2290	2037	2060	49.72	NF* ?	5*?	119	6	1	1	5	4	5	clpA_5/1snp3holes	clpA_5/edge0.0	26.7
KM-115	2680	2637	50.40	NF*	24*	1	6	18	11	19	1	12*	clpA_24/1snp;uvrA_12/1snp	-	58.7
RI-2584	2878	2785	50.82	NF	2	2	1	2	2	2	2	10	0	-	48.8
NY-1468	2494	2382	51.15	NF*	5	7	5	1	1*	1	4	9	pyrG_1/1snp	-	572.9
T13-220	2768	2588	51.68	NF*	19	1	5	116*	2	1	1	10	pepX_116/1snp	-	770.2
NJ-2805	3004	2745	52.25	3	4	1	1	1	1	6	1	7	0	-	114.1
KM-102	3461	3120	52.59	NF*	5*	1	1	1	2	1	1	2*	clpA_5/1snp;uvrA_2/1snp	-	870.2
NY-1587	2034	1685	54.69	59	6	1	5	1	1	7	1	19	0	-	37.3
NY-2539	3344	2738	54.98	NF*	5*	1	1	15*	1	15*	1	10	clpA_5/3snp;pepX_15/1snp;recG_15/1snp	-	811.9
HIS-2951	3309	2590	56.09	NF	10	7	4	6	1	6	1	6	0	-	364.5
CT-1827	3170	2439	56.52	NF*	21*	1	1*	1	1	6	1	7*	clpA_21/2snp;nifS_1/1snp;uvrA_7/1snp	-	94.9
MA-2682	3372	2542	57.02	NF	6	1	5	1	1	6	1	7	0	-	534.9
Bbcap8	28	21	57.14	1*	1*	1	1	1	1	1	1	1	clpA_1/1snp	-	24.0
RI-2597	3063	2266	57.48	NF	14	1	5	1	6	16	1	125	0	-	40.6
Bbcap29	3282	2181	60.08	NF*	8*	1	5	15*	1	6	1	7	clpA_8/1snp;pepX_15/1snp	-	400.2
NH-2426	3385	2226	60.33	NF*	1*	2*	1	2	2*	16	1	10	clpA_1/2snp;clpX_2/1snp;pyrG_2/1snp	-	658.3
Bbcap17	3144	1979	61.37	NF	4	1	1	2	1	6	1	7	0	-	374.4
WI-205	3534	2136	62.33	NF*	8*	2	5	6*	2	8	1	2	clpA_8/1snp;pepX_6/1snp	-	340.4
HIS-3040	2890	1731	62.54	NF*	6	1	1	1	1	7	1	11*	uvrA_11/1snp	-	58.3
NY-2600	2660	1489	64.11	403*	1	1	1	1	1	1	1	11*	uvrA_11/1snp	-	730.3
NS12-24	3311	1701	66.06	NF*	4*	1	1	2	1	16	1	10	clpA_4/1snp	-	106.7
MA-1927	3065	1334	69.67	NF*	4*	1	1	1	1	18	1	1	clpA_4/1snp	-	48.4
CT-1807	3351	1395	70.61	NF*	9*	1	1	116	1	15	1	19*	clpA_9/1snp;uvrA_19/1snp	-	196.0
CT-2493	3255	1349	70.70	3*	4	1	1	1	1	6*	1	7	recG_6/1snp	-	326.4
RI-2669	2465	938	72.44	NF?	1	1	1	1?	1	18	1	7	0	pepX_1/edge2.0	22.4
Bbcap31	2602	555	82.42	1	1	1	1	1	1	1	1	1	0	-	566.3
HIS-2955	1716	144	92.26	1	1	1	1	1	1	1	1	1	0	-	264.6

Table S6. Polymorphic site frequency for sequences derived from single colonies

Sample	Polymorphic	Monomorphic	%Poly
156a	0	3622	0.00
72a	0	3863	0.00
Bb16-105	0	3569	0.00
Bb16-122	0	2227	0.00
Bb16-128	0	4076	0.00
Bb16-133	0	4070	0.00
Bb16-136	0	3849	0.00
Bb16-138	0	4070	0.00
Bb16-139	0	4018	0.00
Bb16-16-1	0	3920	0.00
Bb16-163	0	29	0.00
Bb16-167	0	3934	0.00
Bb16-174	0	3820	0.00
Bb16-186	0	3589	0.00
Bb16-188	0	3506	0.00
Bb16-22-2	0	2202	0.00
Bb16-23-2	0	3870	0.00
Bb16-249	0	3592	0.00
Bb16-268	0	3831	0.00
Bb16-33-3	0	3718	0.00
Bb16-52	0	4098	0.00
Bb16-66	0	3755	0.00
Bb16-74-1	0	2462	0.00
Bb16-80	0	3773	0.00
Bb16-90-2	0	4023	0.00
Bb16-93	0	3836	0.00
CA-11.2A	0	3783	0.00
CA382	0	862	0.00
JD1	0	3547	0.00
PAbe	0	55	0.00
PAli	0	55	0.00
PBre	0	2125	0.00
PDri	0	4046	0.00
PGl	0	2034	0.00
PKif_I	0	4013	0.00
PKif_II	0	4084	0.00
PKu	0	2163	0.00
PSst	0	2144	0.00
WI91-23	0	3821	0.00
Z41293	0	11097	0.00
CA8	1	5628	0.02
Bb16-126	1	4128	0.02
Bb16-145	1	4110	0.02
Bb16-130	1	4065	0.02
Bb16-134	1	4056	0.02
Bb16-71-2	1	4040	0.02
Bb16-17-1	1	4032	0.02

Bb16-132	1	4020	0.02
Bb16-250	1	3952	0.03
94a	1	3933	0.03
Bb16-193-2	1	3865	0.03
Bb16-112	1	3782	0.03
Bb16-60-1	1	3614	0.03
Bb16-175	1	3594	0.03
Bb16-146	1	3585	0.03
Bb16-87	1	3569	0.03
Bb16-182	1	3555	0.03
Bb16-183	1	3521	0.03
Bb16-135	1	2448	0.04
Bb16-47	1	2441	0.04
ZS7	1	2169	0.05
PMeh	1	2102	0.05
64b	1	2066	0.05
N40	2	4119	0.05
SV1	6	12172	0.05
Bb16-178-2	2	3983	0.05
Bb16-55-1	2	3890	0.05
Bb16-202	2	3860	0.05
Bb16-110	2	3828	0.05
Bb16-198	2	3602	0.06
Bb16-142	2	3591	0.06
Bb16-170	2	3580	0.06
Bb16-184	2	3545	0.06
Bb16-181	3	4099	0.07
Bb16-150	3	4070	0.07
Bb16-15-2	3	3898	0.08
Bb16-164	3	3862	0.08
Bb16-54	3	3843	0.08
118a	3	3815	0.08
Bb16-178-1	3	3583	0.08
Bb16-149	3	3570	0.08
PLue	3	3346	0.09
PFi_I	2	2193	0.09
29805	4	3919	0.10
Bb16-57-1	4	3637	0.11
Bb16-10-2	4	3561	0.11
Bb16-85	5	4064	0.12
PFhe_II	3	2205	0.14
Bol26	3	2127	0.14
Bb16-111	6	3626	0.17
Bb16-62	6	3579	0.17
PFhe_I	4	2204	0.18
Bb16-59-1	7	3772	0.19
Bb16-49	19	3484	0.54
